# Utilizing nullomers in cell-free RNA for early cancer detection

**DOI:** 10.1038/s41417-024-00741-3

**Published:** 2024-02-14

**Authors:** Austin Montgomery, Georgios Christos Tsiatsianis, Ioannis Mouratidis, Candace S. Y. Chan, Maria Athanasiou, Anastasios D. Papanastasiou, Verena Kantere, Nikos Syrigos, Ioannis Vathiotis, Konstantinos Syrigos, Nelson S. Yee, Ilias Georgakopoulos-Soares

**Affiliations:** 1 Institute for Personalized Medicine, Department of Biochemistry and Molecular Biology, The Pennsylvania State University College of Medicine, Hershey, PA USA; 2 School of Electrical and Computer Engineering, National Technical University of Athens, Athens, Greece; 3 Institute for Human Genetics, University of California San Francisco, San Francisco, CA USA; 4 Department of Biomedical Sciences, University of West Attica, Athens, Greece; 5 Third Department of Internal Medicine, Sotiria Hospital, National and Kapodistrian University of Athens, School of Medicine, Athens, Greece; 6 Next Generation Therapies Program, Penn State Cancer Institute; Division of Hematology-Oncology, Department of Medicine, Penn State Health Milton S. Hershey Medical Center, Hershey, PA USA

**Keywords:** Biomarkers, Cancer

## Abstract

Early detection of cancer can significantly improve patient outcomes; however, sensitive and highly specific biomarkers for cancer detection are currently missing. Nullomers are the shortest sequences that are absent from the human genome but can emerge due to somatic mutations in cancer. We examine over 10,000 whole exome sequencing matched tumor-normal samples to characterize nullomer emergence across exonic regions of the genome. We also identify nullomer emerging mutational hotspots within tumor genes. Finally, we provide evidence for the identification of nullomers in cell-free RNA from peripheral blood samples, enabling detection of multiple tumor types. We show multiple tumor classification models with an AUC greater than 0.9, including a hepatocellular carcinoma classifier with an AUC greater than 0.99.

## Introduction

Cancer is characterized by the accumulation of somatic mutations and uncontrolled clonal proliferation of malignant cells [1]. Though there have been important advances in cancer therapeutics, cancer remains the second leading cause of death worldwide [2]. The vast majority of malignant tumors are detected at a late stage, where the likelihood of survival declines steeply [3]. Early cancer detection is associated with improved clinical outcomes [4]. Therefore, there is a need for novel biomarkers to facilitate early cancer detection as well as surveillance at the population level.

Cancer biomarker development has involved proteomic, transcriptomic and metabolomic profiling, DNA methylation, circulating tumor cells, and cell-free DNA (cfDNA) [5–9]. However, these methods have been shown to have suboptimal sensitivity and specificity. There is sufficient evidence that cancer cells release cfRNA, which can be detected in the blood [10]. cfRNA represents a highly dynamic biomarker, since it can indicate expression changes in real time. Importantly, highly expressed tumor-associated genes can be over-represented in cfRNA samples relative to their lower frequency in cfDNA. cfRNA can also provide information about the tissue of origin as there are tissue-specific and cancer-specific transcriptomic differences [10]. Consequently, cfRNA can provide information that is complementary to that derived from cfDNA and could prove particularly useful for tumors with lower mutational load.

Kmers, which are contiguous sequences of length k composed of nucleotides in genomics or amino acids in proteomics. Nullomers are the shortest sequences that are absent from the human genome [11, 12]. By extension, nullpeptides are peptides that are absent from the human proteome. We also recently examined the shortest sequences unique to a species, termed quasi-primes [13, 14]. We and others have previously genomically characterized nullomers and provided evidence for negative selection constraints and for emergence due to germline variants [15, 16]. In cancer cells, a set of nullpeptides have been shown to elicit cytotoxic activity, impact the tumor immune microenvironment and affect the tumor transcriptome [17]. For example, nulpeptides 9 R and 9S1R demonstrate extensive effectiveness against various cancer types, indicating their potential as promising therapeutic agents in cancer treatment [17, 18]. Nullpeptides can also emerge due to somatic mutations in cancer [19]. Recently, we have also investigated the relevance of nullomers in cancer; by analyzing more than 2,700 Whole Genome Sequenced primary tumors we provided evidence for the emergence of nullomers during cancer development while also showing the effectiveness of nullomers as early cancer detection biomarkers using cfDNA [20]. Even though exonic regions are enriched for mutations that cause nullomer emergence, it is still unclear whether nullomers in cfRNA can be used for the early detection of cancer or carry prognostic and/or predictive relevance.

Along these lines, we were interested to examine nullomers’ utility as novel cfRNA biomarkers for early cancer detection. Here, we perform an extensive analysis of nullomer emergence across more than 10,000 Whole Exome Sequencing (WES) matched tumor-normal samples [21]. We evaluate the distribution of nullomer emergence events across tumor types and patients and identify recurrent nullomer emergence events within cancer genes. Finally, we use cfRNA data obtained from liquid biopsy samples to detect cancer using nullomers. Our findings provide evidence for the utility of nullomers as cancer diagnostic biomarkers in cfRNA.

## Results

### Mutation type preferences during nullomer emergence in cancer

**Fig. 1:**
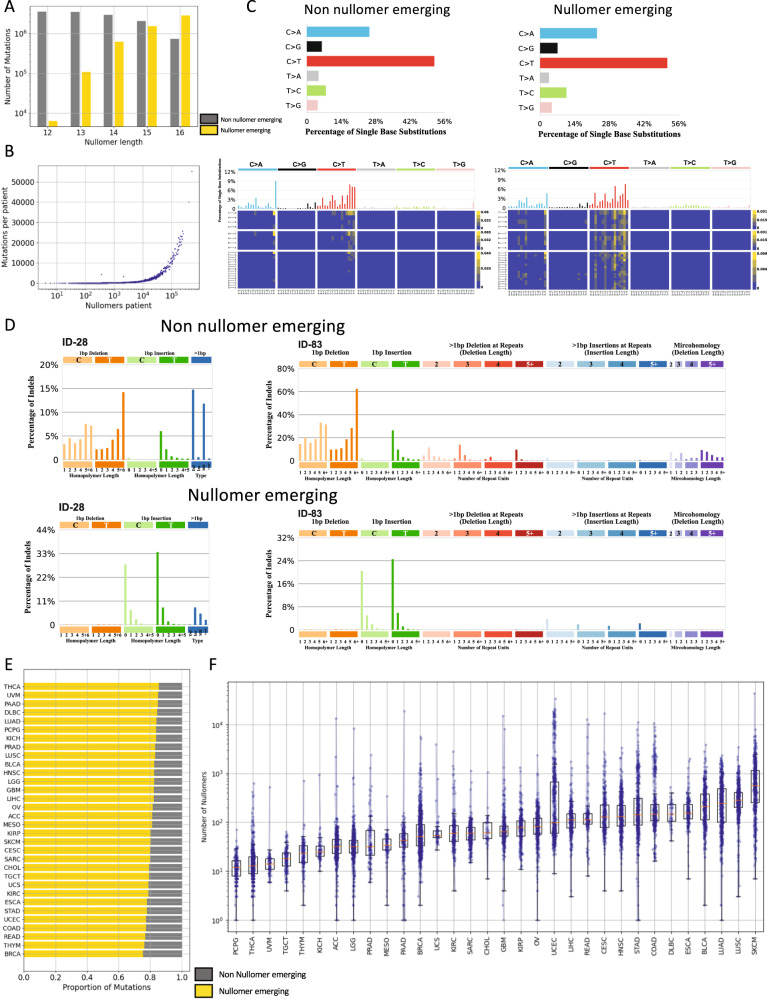
Characterization of nullomer emergence in WES patient samples. **A** The number of mutations causing nullomer emergence (in yellow) relative to those that do not cause nullomer emergence for kmer lengths between 12 bp and 16 bp. **Β** Association between the number of WES somatic mutations and the number of nullomers that emerge per patient. Results shown for sixteen-mer nullomers. **C** Proportion of substitution types in nullomer emerging and non-nullomer emerging substitutions for sixteen bp nullomer length. Characterization of differences in substitution type preference for non-nullomer and nullomer emerging mutations using the 96 substitution type channels. **D** Characterization of differences in indel preference for non-nullomer and nullomer emerging mutations using the 28 and 83 indel mutation channels. **E** Proportion of mutations (in yellow) causing sixteen bp nullomer emergence across cancer types. The proportion of mutations that do not cause nullomer emergence are shown in gray. **F** Number of nullomers detected for each cancer patient in each cancer type for 16 bp nullomer length. Every dot represents a patient.

Even though nullomer sequences are absent from the human genome, somatic mutations can cause the emergence of nullomers during cancer development. We first identified nullomers across kmer lengths of up to 16 base-pairs (bp) long for the reference human genome as previously described in [20]. We analyzed mutation data from over 10,000 WES matched tumor-normal pairs across 32 cancer types to detect emergence of nullomers due to somatic mutations. Germline mutations were removed using the tumor-normal pairs and, thus, did not affect the analysis. The total number of different sixteen bp nullomers that emerged across all somatic mutations in this cohort was 29,774,302, representing 0.69% of the 16 bp kmer space. Moreover, we found that the proportion of somatic mutations that cause nullomer emergence increased from 0.178% at 12 bp kmer length, to 79.76% at 16 bp kmer length (Fig. 1A). This finding indicates that the majority of exonic somatic mutations cause the emergence of one or more nullomers at longer kmer lengths.

We also report a strong correlation between the number of mutations and the number of emerged nullomers across patients with cancer (Pearson correlation, *r* > 0.98, *p*-value < 0.0001 across kmer lengths; Fig. 1B, Supplementary Fig. 1). In addition, the average number of nullomers that emerge by each individual mutation increased with the nullomer length (Supplementary Fig. 2). We also examined how different mutation types affect the likelihood of a nullomer emergence. There were significant differences in which of the substitution mutations across the 96 possible trinucleotide changes gave rise to nullomers and which did not (Fig. 1C). For example, we observed that nullomer emerging mutations show a smaller proportion being TCT > TAT and a larger proportion being GCG > GTG than non-nullomer emerging mutations (Fig. 1C). We also explored indels and doublet base substitutions for nullomer emergence. We found that mononucleotide repeat tract deletions almost never cause nullomer emergence; rather, nullomer emergence occurred primarily at 0 bp or 1 bp homopolymer length insertions (Fig. 1D, Supplementary Fig. 3). These findings indicate that the mutation type significantly influences the likelihood of nullomer emergence.

### Identification of nullomer emergence across 10,000 WES tumor samples

We investigated how kmer length affected the proportion of mutations which cause the emergence of nullomers across individual cancer types. The proportion of mutations which caused nullomer emergence was extremely small at twelve and thirteen bp lengths (Supplementary Fig. 4a, b), whereas at sixteen bp lengths the majority of somatic mutations caused nullomer emergence across cancer types (Fig. 1E, Supplementary Fig. 4). We also report differences in the proportion of mutations causing nullomer emergence between cancer types. Across multiple kmer lengths, thyroid cancer (THCA) and breast cancer (BRCA) had the highest (85.55% for 16 bp) and lowest (75.50% for 16 bp) proportion of mutations causing nullomer emergence, respectively (Fig. 1E, Supplementary Fig. 4).

Next, the number of nullomers identified across individual cancer types and patients was explored. The mean number of nullomers identified across patients ranged between 0.62 and 278.5 for 12 bp and 16 bp kmer lengths, respectively. The cancer types with the highest and lowest number of nullomers emerging per patient were skin cutaneous melanoma (SKCM) and pheochromocytomas and paragangliomas (PCPG), respectively (Fig. 1F). We also observed one extreme case in which one patient produced 508,100 nullomers, indicating a hypermutator phenotype. We conclude that nullomer emergence occurred for a significant fraction of somatic mutations across cancer types, when examining kmer lengths of fourteen bps or higher.

### Nullomer emergence across cancer genes

**Fig. 2:**
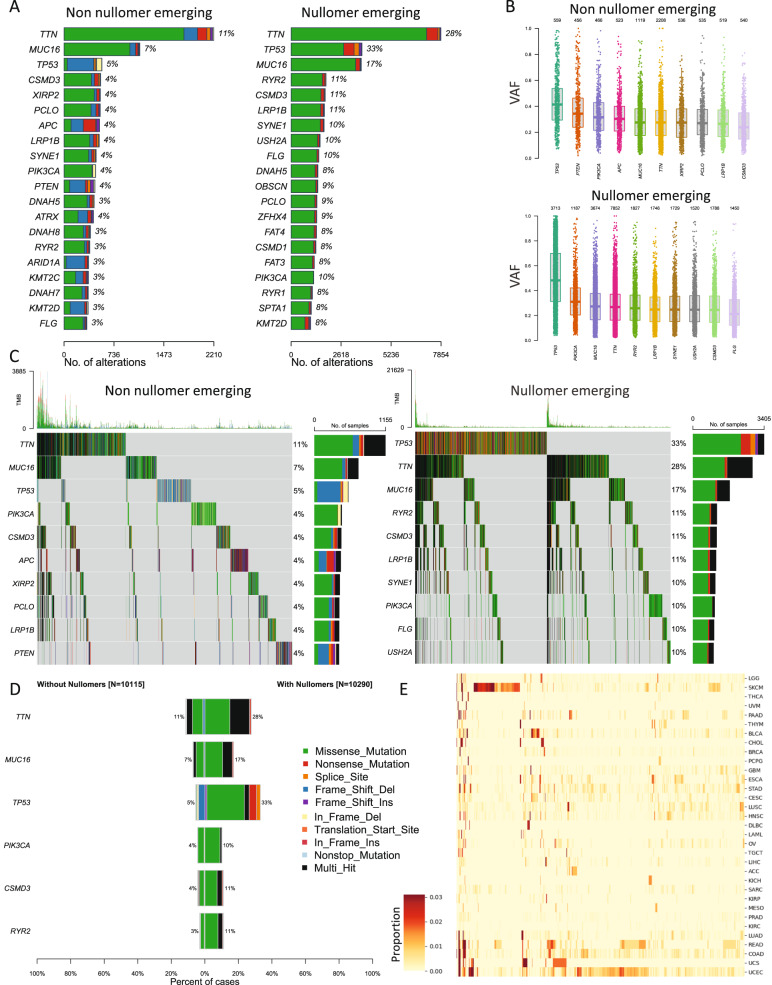
Identification of nullomer emergence across cancer genes. **A** Percentage of patients with each mutated gene across cancer types for the top twenty most mutated genes. **B** Variant allele frequency of mutations that do not cause sixteen-mer nullomer emergence and of mutations that cause sixteen-mer nullomer emergence. **C**, **D** Number of mutations in top cancer genes for mutations that either do not cause or cause nullomer emergence. **E** Proportion of patients in which each of the top 16 bp top 10,000 nullomers from across all patients is found.

Subsequently, we compared the frequency of nullomer emerging mutations and non-nullomer emerging mutations across genes. Firstly, across the most frequently mutated genes in the patient cohort, we identified differences between the set of mutations that did not cause nullomer emergence and those that did. For instance, *TP53* was more frequently found to have nullomer emerging mutations relative to other cancer genes, and those nullomer emerging mutations were primarily missense mutations (Fig. 2A). Interestingly, the variant allele frequency was higher in *TP53* for mutations that caused nullomer emergence (Fig. 2B). Similar results were also obtained for other cancer genes such as *RYR2* (Fig. 2A), indicating biases in the frequencies between mutations that did or did not cause nullomer emergence across patients.

We observed that for sixteen bp nullomers, more somatic mutations caused nullomer emergence than those that did not across the top cancer genes (Fig. 2B, C). For instance, 33% of patients had nullomer emerging mutations at *TP53*, whereas only 5% had mutations that did not cause nullomer emergence in the same gene (Fig. 2B–D). We also found that the types of mutations which caused nullomer emergence in the most frequently mutated cancer genes were different from those which did not cause nullomer emergence and were primarily missense, nonsense and multi-hit mutations (Fig. 2B–D). Thus, it can be inferred that there is nullomer emergence associated with the vast majority of mutations in cancer genes are mutated. Significant differences were detected in the frequency of nullomer emergence from somatic mutations between cancer types across kmer lengths (Fig. 2E; Supplementary Fig. 6). Finally, we performed an analysis examining the density of nullomer emerging mutations in coding regions across genes. We find that the genes with the highest density include *TP53*, *KRAS* and *CDKN2A* among others (Supplementary Fig. 7). These nullomer signatures could be used in liquid biopsy as additional cancer biomarkers.

### Nullomer emergence at mutational hotspots

**Fig. 3:**
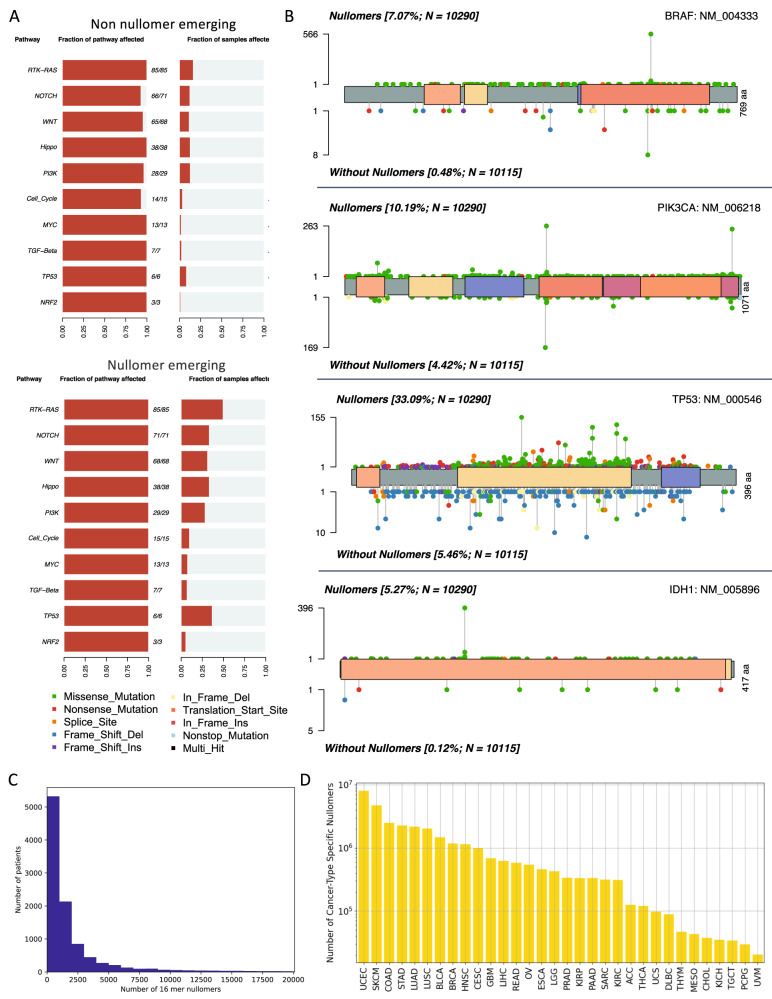
Identification of highly recurrent nullomers across cancer types and patients. **A** Frequency with which genetic pathways were affected for mutations that do not cause or cause nullomer emergence. **B** Lollipop plot displaying mutation distribution for nullomer emerging and non-nullomer emerging mutations. **C** Number of patients in which each of the top sixteen-mer nullomers was detected. **D** Number of cancer-type specific nullomers across all cancer types examined at length sixteen.

For the most mutated cancer genes, we compared the distribution and frequency of nullomer emerging mutations to those that did not cause nullomer emergence across the length of each gene. Across the genetic pathways involved in cancer, we found that nullomer emerging mutations are more common than mutations that do not cause nullomer emergence (Fig. 3A), which is consistent with the majority of mutations causing nullomer emergence at length sixteen (Fig. 1A). When examining individual cancer genes, we observed that there were loci at which nullomers repeatedly emerged (Fig. 3B**;** Supplementary Table 1) and these loci represented cancer driver events. Oncogenes, such as *BRAF*, *PIK3CA* and *IDH1*, showed individual nullomer emerging hotspots, whereas tumor suppressors such as *TP53* showed dispersed patterns of nullomer emergence across the gene body (Fig. 3B). Thus, the characterization of nullomer emergence across individual cancer genes can enable sample classification based on clinical targets and inform on the biological effect of a mutation.

Nullomers that emerge recurrently across multiple cancer patients are more likely to be predictive of the tissue of origin of a cancer. We therefore examined how frequently each nullomer emerged in multiple patients across all the considered cancer types or at individual cancer types. We report that most nullomers are not recurrent; however, a small subset can be detected with high frequency across cancer patients (Fig. 3C). We also showed that the most recurrently emerging nullomers are primarily found at a single cancer gene within a particular locus and primarily involve known driver mutations (Table 1). For instance, the most recurrently observed nullomer was found at *BRAF* across 5.5% of cancer patients, while other top emerging nullomers were found at individual loci in *IDH1*, *PIK3CA*, *KRAS* and *TP53* (Table 1), all of which are known cancer genes.

**Table 1:** Selection of cancer genes and the corresponding most recurrently emerging 16 bp nullomers across cancer types and patients.

16 bp nullomer	Gene	Percentage of patients	Number of cancer types
CTCCATCGAGATTTCT	*BRAF*	5.5%	11
CTATCATCATAGGTCA	*IDH1*	3.48%	6
TGAAATCACTAAGCAG	*PIK3CA*	2.56%	19
TCTTGCCTACGCCATC	*KRAS*	1.93%	18
GGAGGTTGTGAGGCAC	*TP53*	1.51%	19

Selection is based on locus-specific nullomers.

Interestingly, we identified a second set of highly recurrent nullomers, which are observed in multiple cancer genes (Table 2). The top recurrent nullomers observed are found in clusters of paralogous genes. For instance, “CTCCAGTGTGAGTTAT” was found to emerge across 34 genes, most of which were zinc-finger genes. Additionally, “GTTGTTCTCGCGGACA” was found in 13 genes, all of which were different members of the Protocadherin Beta gene family. Therefore, highly recurrent nullomers can be identified across WES tumor samples and can be potentially utilized for the early detection of cancer with liquid biopsies.

**Table 2:** Selection of cancer genes and the corresponding most recurrently emerging 16 bp nullomers across cancer types and patients.

16 bp nullomer	Number of genes	Percentage of patients	Number of cancer types
CTCCAGTGTGAGTTAT	34	0.456%	9
GTTGTTCTCGCGGACA	13	0.379%	14
CACCGCCACAAACAGG	15	0.369%	9
TGGCCTATGATTGTTA	7	0.262%	7

Selection is based on nullomers detected across multiple loci.

### Identification of cancer-type specific nullomers

We were also interested in investigating if certain nullomers appear in individual cancer types but are otherwise absent from all other cancer types and are, therefore, cancer-type specific. We identified cancer-type specific nullomers across all the cancer types examined (Fig. 3D), with the highest number of cancer-type specific nullomers being observed in uterine corpus endometrial carcinoma (UCEC), SKCM and colorectal adenocarcinoma (COAD), three of the cancer types with the highest mutational burden. We found that at longer kmer lengths, the number of cancer-type specific nullomers being identified increased (Supplementary Fig. 8).

### Identification of nullomers in cfRNA for cancer detection

We examined if the identified nullomers can be used to detect cancer in liquid biopsies using cfRNA data. We performed our analyses using two datasets that encompassed lung, colorectal, stomach, esophageal and liver cancers, as well as healthy controls [22, 23]. For each sample, we identified the nullomers present for nullomer lengths between 14 bp and 16 bp and generated classification models to estimate our ability to detect cancer. The nullomers that we incorporated in this analysis were the top 100,000 most frequently emerging nullomers across all cancer types (Fig. 1), thus serving as a general list of nullomers to detect multiple cancer types.

**Fig. 4:**
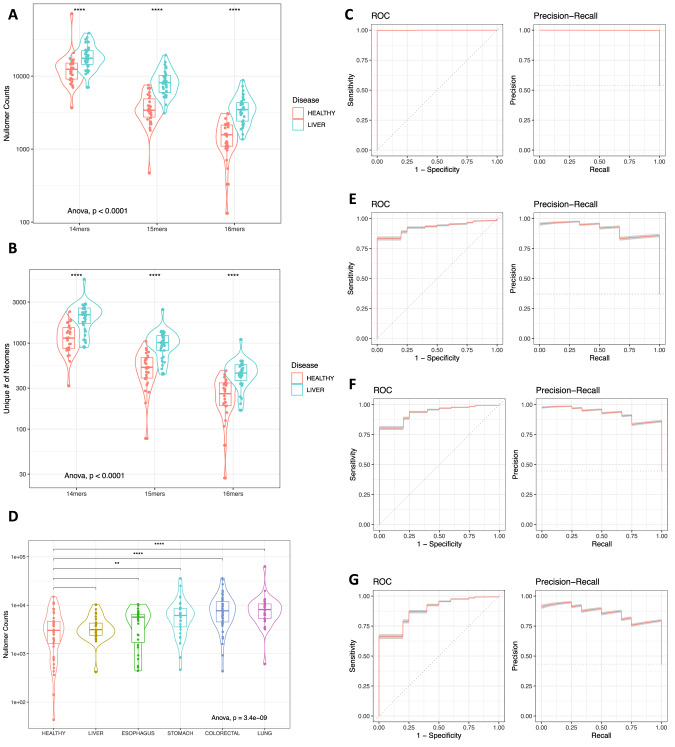
Identification of nullomers in cfRNA derived from liquid biopsy samples for early cancer detection. **A** Counts of nullomers identified in healthy and HCC samples using 14mer, 15mer and 16mer nullomers. Samples are grouped by disease state. **B** Number of unique nullomers identified in healthy samples and HCC. Results shown for 14mer, 15mers and 16mer nullomers. **C** ROC curve and precision recall for liver cancer. **D** Counts of nullomers identified in healthy and cancer samples for liver, esophageal, stomach, colorectal and lung cancers using 16mer nullomers. Cancer samples are grouped by cancer type. **E**–**G** ROC curve and precision recall for (**E**) liver cancer, (**F**) stomach cancer and (**G**) lung cancer.

For the first dataset, which encompassed hepatocellular carcinoma (HCC) and healthy control data, we examined the frequency of nullomer emergence in cancer relative to controls [22]. We observed that the total counts of nullomers detected in cfRNA derived from liquid biopsies of HCC patients was significantly higher than for the healthy controls (Welch Two Sample *t* test, *p*-value < 0.0001 across kmer lengths; Fig. 4A). Next, we examined if the size of the set of unique nullomers differed between the two groups and found consistent patterns (Welch Two Sample *t* test, *p*-value < 0.0001 across kmer lengths; Fig. 4B). We also trained a machine learning model to examine if we can accurately detect HCC based on the nullomers identified in each sample. We generated a lasso logistic regression classification model which was able to detect cancer samples in all cases (AUC = 1; Fig. 4C). Results were highly consistent with different kmer lengths, and we were able to accurately detect HCC also using fourteen (AUC = 0.999) and fifteen (AUC = 0.998) bp nullomer lengths (Supplementary Fig. 9). In addition to good discrimination between HCC and healthy samples, each model showed accurate probabilistic predictions as evidenced by a Brier score less than or equal to 0.02 (Supplementary Fig. 10). We also examined the top most informative features and observed that the most informative nullomers were found at liver cancer-associated genes, including *FTH1*, *EEF2*, *TMSB10*, *ACTB* and the long non-coding RNA *MALAT* among others (Table 3). Our findings provide evidence for the utility of nullomer identification in cfRNA for cancer detection.

**Table 3:** Selection of the most informative nullomers for detection of liver cancer.

Gene	Nullomer	Proportion	Reference
*ACTB*	AAGGCCAACCGCAAGA	0.992	[39]
*BAC RP11-96H19*	ATCAGCAAGCACACCA	0.958	[40]
*EEF1A1*	CCAATGGAAGCCGC	1.00	[41]
*EEF2*	CTGGCGTAGAGGCAGC	1.00	[42]
*FTH1*	CGGCCGCCCATAGTCA, ATGACGACTGCGTCC, CGACTGCGTCCACC	0.999, 0.999, 0.965	[43]
*MALAT1*	GAAGTTTGCAGTGGAA	0.954	[44]
*RPL32*	ACCAATGTTGGGCATG	0.942	[45]
*TMSB10*	GATTGGGGGGGGGCCC, ATTGGGGGGGGGCCC	1.00, 0.913	[46]

Next, we examined a dataset that included liquid biopsy-derived cfRNA data from liver, esophageal, stomach, colorectal and lung cancers, as well as healthy control cfRNA data [23]. We found that, on average, samples from each cancer type displayed more nullomer counts than the controls (Fig. 4D, Welch Two Sample *t* test, *p*-value = 0.0001491), suggesting that nullomers can indeed be used to differentiate between cancer patients and healthy controls across disparate cancer types. Next, we also created lasso logistic regression classification models for cancer detection and examined their performance for each cancer type. The classification models for liver, stomach, and lung cancer had an AUC of 0.922, 0.927, and 0.877, respectively. We also found the most informative nullomers for each of the different cancer classification models (Table 4). The reported results indicated the models’ ability to accurately classify cancer and healthy samples across different cancer types (Fig. 4D–G) with particularly high performance for stomach, thus revealing the potential of RNA nullomers to facilitate early cancer detection.

**Table 4:** Selection of the most informative nullomers for detection of multiple cancer types including stomach, lung, colorectal, esophageal and liver cancers.

Gene	Nullomer	Cancer Type	Proportion	Reference
*ACRBP*	AAACTGGCCTAGAGTC	Stomach	0.954	[47]
*ACTB*	AAGGCCAACCGCAAGA	Stomach	1.00	[39]
CAVIN2	GGAAAGCCTGCACACC	Lung	0.999	[48]
*FTH1*	CGGCCGCCCATAGTCA	Stomach, Colorectal	0.986, 0.973	[43]
*KIF2A*	GGCGGAAAAGGCGGGA	Stomach	0.908	[49]
*MALAT1*	GAAGTTTGCAGTGGAA	Esophagus	0.958	[44]
*MPC1*	GGCCACCCCCCCGGCA	Esophagus	0.931	[50]
*SNX3*	GAGACCGTGGCTGGCA	Stomach	0.905	[51]
*UBC*	CATCGAGAATGGCAAG	Stomach	0.994	[52]

## Discussion

In this study, we have characterized nullomer-emergence across more than 10,000 WES tumors in 32 cancer types and investigated their utility as cancer biomarkers in liquid biopsies with cfRNA. The usage of a cfRNA-based cancer detection assay offers several advantages. For instance, the process by which tumor-derived RNA is introduced into the bloodstream likely exhibits differences from cfDNA, including its transfer with exosomes [10, 24]. In addition, the usage of cfRNA in diagnostics can incorporate overall expression levels and dynamic expression changes. By utilizing nullomers, we reduce the needed biological material to detect mutations. Thus, using nullomers within cfRNA should increase the sensitivity of identifying mutations from the matched tumor. Increasing sensitivity is crucial for uncovering both tumors with low mutational burden and mutations with a low allelic fraction. An example of where increased sensitivity is needed is hepatocellular carcinoma, where traditional cfDNA analysis methods are only able to recover 19.5–43% of mutations found on tumor biopsy [25–27].

In contrast to the usage of nullomers in WGS tumor samples, in which most identified nullomers are non-coding and are passenger mutations [20], in WES we observe a substantial fraction of emerging nullomers being cancer drivers and actionable targets. We also provide evidence for the usage of nullomers in cfRNA for cancer detection across multiple cancer types. We previously described the emergence of nullomers due to putative mutations and germline variants [15]. The number of nullomers emerging from somatic mutations increases exponentially as a function of kmer length, which aligns with the previous findings for putative and germline variants. This is expected based on the number of possible kmers for a given length; we observe that this property enables us to capture a larger proportion of the somatic mutations (Supplementary Fig. 4; Fig. 1E), which in turn results in improved cancer detection (Fig. 4A, B, D). Our hepatocellular carcinoma nullomer model shows higher performance than previously used detection panels of ncRNAs [22, 28, 29]. Additionally, our model shows better performance than models which were trained on somatic copy number aberrations [30]. To further show the information gain provided by nullomers, we plan to compare the results of the nullomer models of cfRNA of patients with cancer to the same model applied to normal sequences subjected to random in-silico mutation.

It is important to note that these results are limited by the sample size of the datasets. Larger cohort sizes with information about cancer staging are needed to validate the use of specific nullomers in models for cancer detection before one day making it to clinical trial. In future work, we plan to incorporate additional disparate cancer types to characterize the performance of our nullomer-based approach between them. It will be of interest to directly compare the performance of predictive models using cfDNA and cfRNA for the same patients as well as their integration into multi omics predictive models. To account for functional mutations, it may also prove fruitful to incorporate the predicted protein sequences of the cfRNA into the predictive models. Furthermore, as immunotherapies and personalized treatments are advancing, nullomer based cfRNA-based diagnosis could be coupled with the identification of neoantigens for personal cancer vaccine development or other patient-tailored therapies.

Therefore, in future work, we envision an integrated setting in which we can use nullomers across the stages of cancer care including cancer detection, diagnosis and treatment choice. Finally, cfRNA biomarkers can be combined with DNA-based, protein-based and other cancer biomarkers to improve and advance the early diagnosis of cancer.

## Methods

### Mutation dataset

Whole exome sequencing mutation data from tumor samples with matched controls were downloaded from https://api.gdc.cancer.gov/data/1c8cfe5f-e52d-41ba-94da-f15ea1337efc for over 10,000 whole exome sequencing tumor samples spanning 32 cancer types, from The Cancer Genome Atlas. Throughout the study, the GRCh37 reference human genome was used unless otherwise stated.

### Nullomer emergence from somatic mutations

Nullomers were identified as previously described in [15]. Nullomer emergence was performed for kmer lengths of 12–16 bp for each somatic mutation across cancer patients and tumor types. Somatic mutations were separated into nullomer emerging and mutations that did not cause nullomer emergence. Maftools was used for the analysis of somatic mutations across cancer genes and at individual loci of specific cancer genes. The density of mutations causing nullomer emergence was estimated at coding regions. Genes were ranked based on the density of nullomer emerging mutations at coding regions (Supplementary Fig. 7).

### Identification of cancer-type specific nullomers

Tumor types were clustered based on the proportion of nullomers shared. Cancer-type specific nullomers represented nullomers that emerged in at least one patient within a cancer type and which were absent from every patient across all other cancer types.

### cfRNA dataset processing

Liquid biopsy cfRNA fastq files were downloaded [22, 23]. Sequences containing the top 100,000 emerging nullomers (12 bp to 16 bp) across cancer types were extracted from each sample’s respective fastq files with BBTools bbduk.sh [31]. The resulting reads were then trimmed with Trim Galore [32] to remove poor quality bases and adapter sequences. The reads were then filtered with BBTools seal.sh [33] to remove common microbial contaminants, UniVec, ERCC spike-in, and ribosomal sequences. The remaining reads were subsequently deduplicated with BBTools dedupe.sh [33]. Aligning was done with BBTools bbmap.sh [33] with stringent parameters (minid = 0.9 kfilter = 25) against a custom genome including GRCh38, SILVA SSU Ref NR 99, and common human viruses. Jellyfish [34] was used to count occurrences of each nullomer in the aligned SAM files.

### Classification model to detect cancer patients from cfRNA

A count matrix of samples by nullomers was used as the starting input for each model. Any counts less than or equal to two were set to zero to decrease false positive counts. Nullomers which had a sum of counts across samples less than 10 were removed. The count matrix was then CPM normalized with edgeR [35]. Samples for each matrix contained healthy samples and samples of a specific cancer type. The R caret package [36] was used to tune the lambda parameter of an L^1^ regularized logistic regression model across twenty values between zero and one. Ten-fold cross-validation was repeated 100 times to detect the best model and evaluate its stability. Models were calibrated with the val.prob function in the rms R package. Models which showed a sigmoid curve between predicted probability and actual probability were then recalibrated with Platt scaling. The R precrec package [37] was used to generate the Precision-Recall and ROC curves based on the repeated cross-validation predictions. Feature importance was assessed using the R glmnet package [38] to perform 10-fold cross-validation repeated 100 times with the previously ascertained lambda parameter. Nullomers with non-zero coefficients were tracked across each of the 1000 models. Nullomers which occurred in over 90% of the models were deemed important and stable across models.

### Supplementary information

Supplementary Material

## Data Availability

The code for this work can be found at: https://github.com/Georgakopoulos-Soares-lab/cfRNA-nullomer-analysis.
