# Supplementary material for: Utilizing nullomers in cell-free RNA for early cancer detection
Source: Cancer Gene Ther. 2024 Feb 14;31(6):861–70. doi: 10.1038/s41417-024-00741-3 (PMC11192629; doi:10.1038/s41417-024-00741-3)
Supplement: Supplementary file 1 — Supplementary Material [file 41417_2024_741_MOESM1_ESM.docx]

**Supplementary Material**

**A B**

**
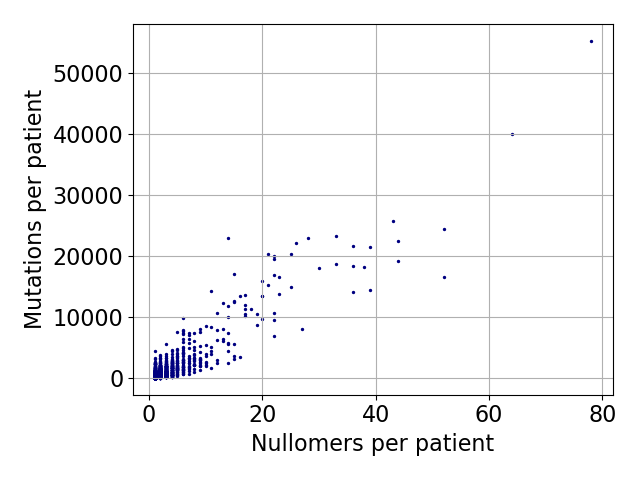

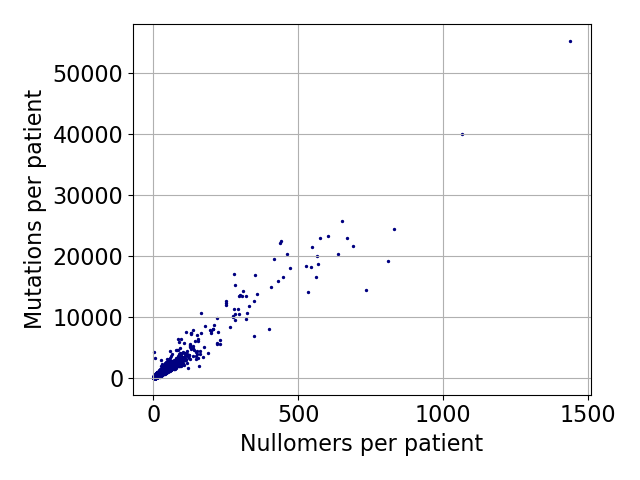
**

**C D**

**
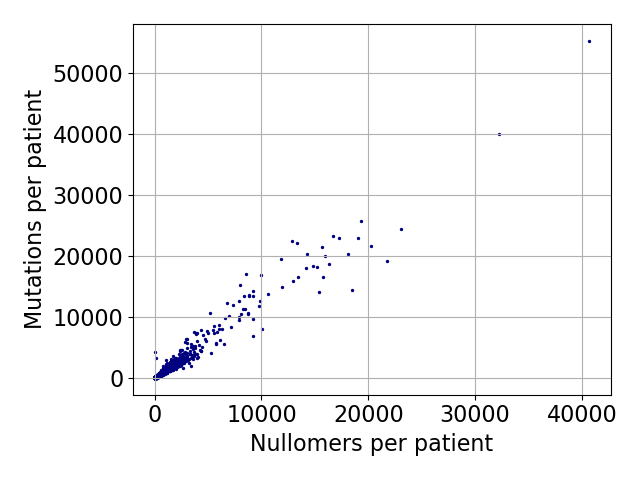

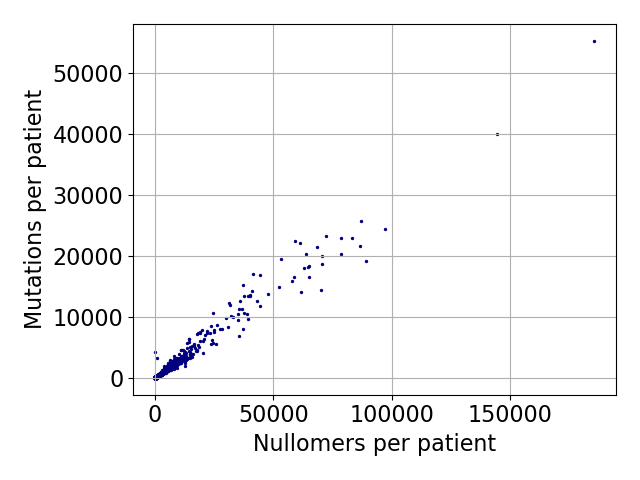
**

**Supplementary Figure 1: Association between the number of protein-altering mutations and the number of nullomers that emerge per patient.** Results shown for **A.** twelve-mer nullomers and **B.** thirteen-mer nullomers, **C.** fourteen-mer nullomers, **D.** fifteen-mer nullomers. In A-D every dot represents a patient sample.

**A B**

**
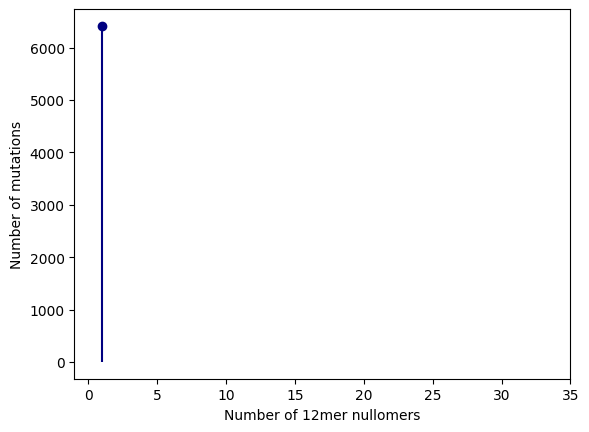

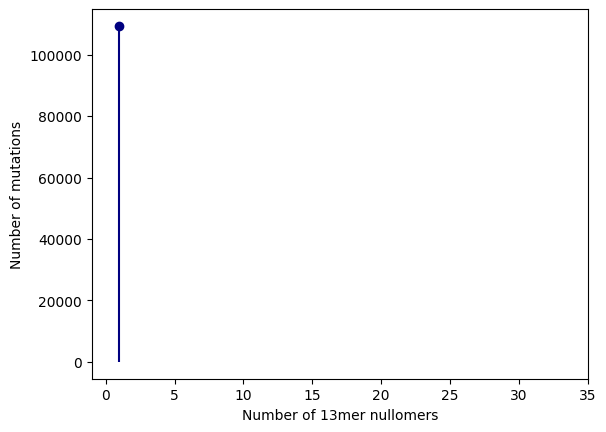
**

**C D**

**
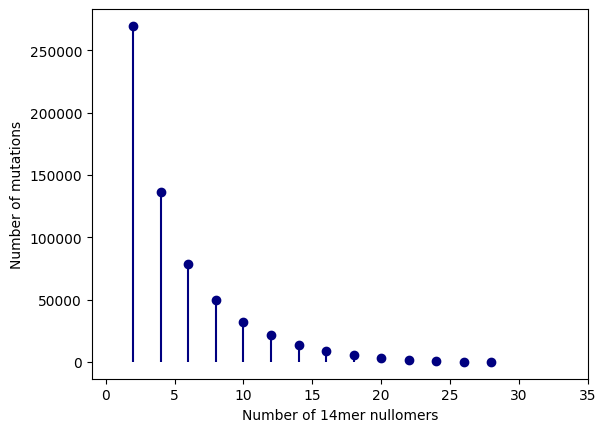

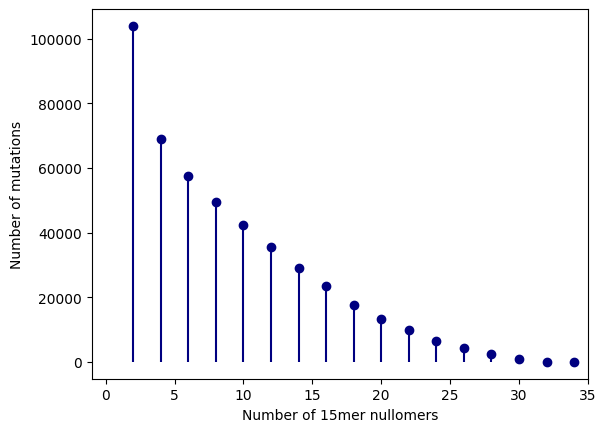
**

**E**

**
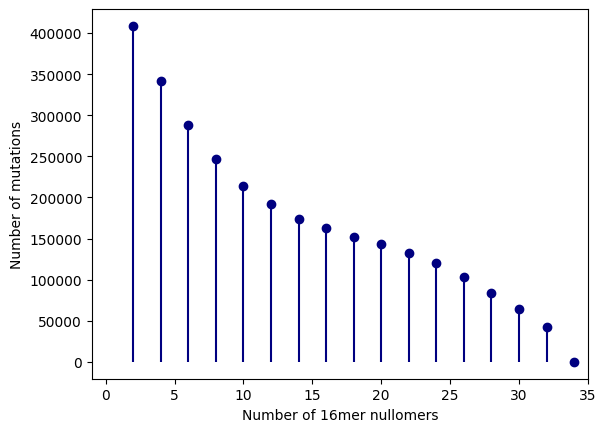
**

**Supplementary Figure 2: Number of nullomers produced from every mutation A.** twelve-mer nullomers and **B.** thirteen-mer nullomers, **C.** fourteen-mer nullomers, **D.** fifteen-mer nullomers. **E.** sixteen-mer nullomers.

**
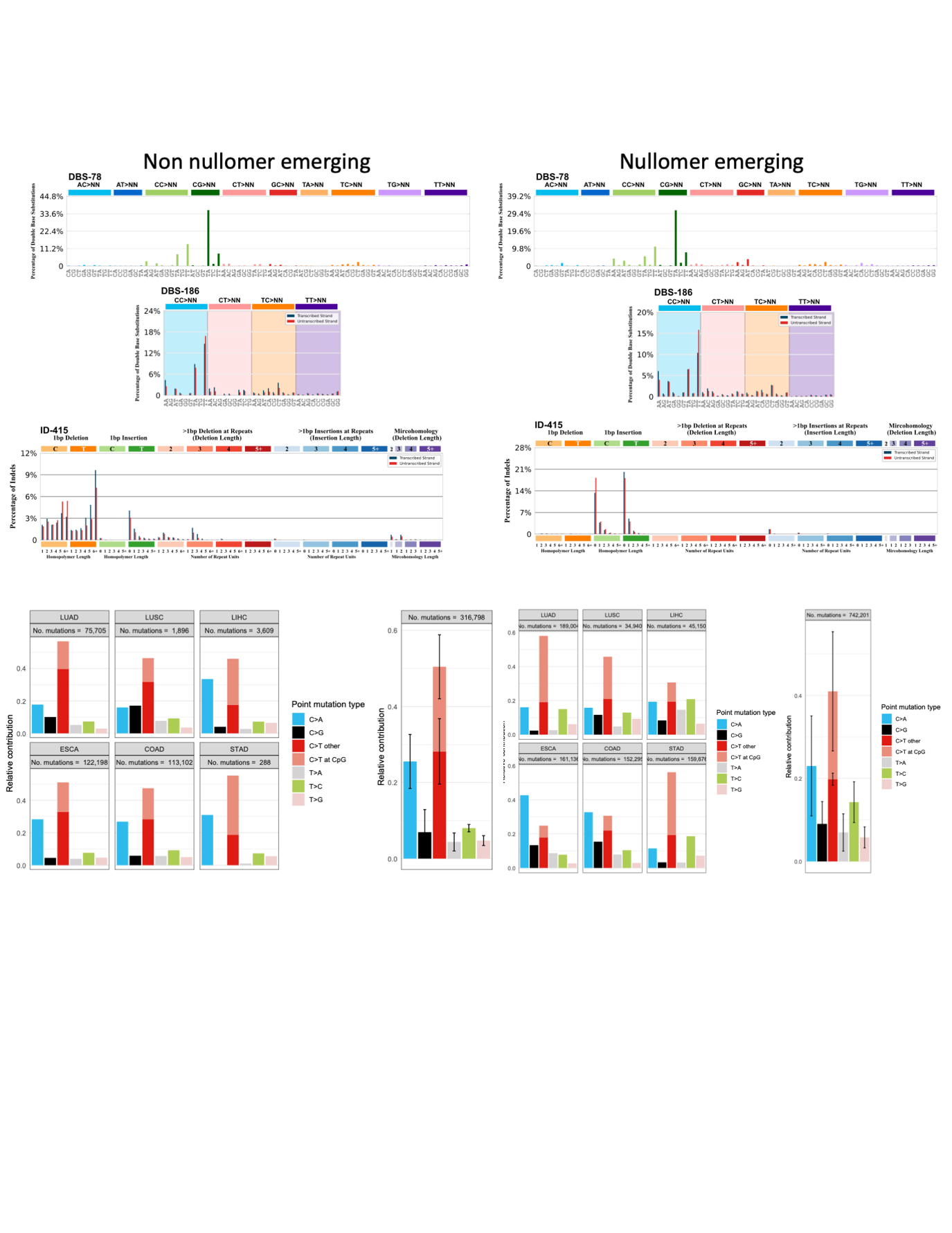
Supplementary Figure 3: Mutational profile of doublet base substitutions (DBS) and indel (ID) mutations.** Results shown for mutations that do not cause nullomer emergence (left) or those that cause nullomer emergence (right).

**A**

**
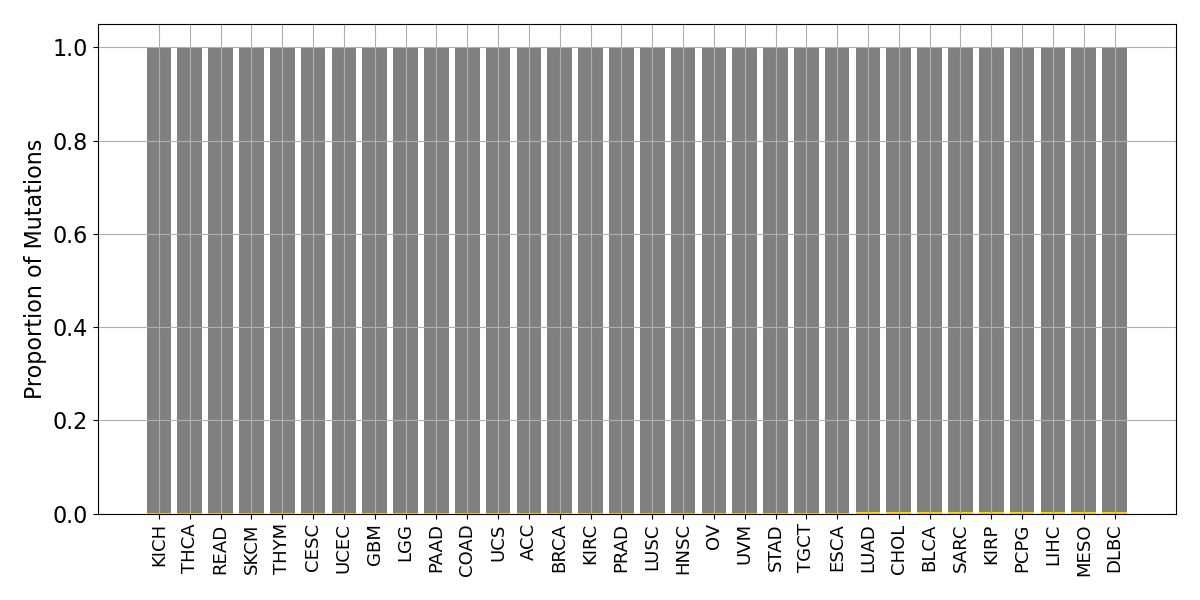

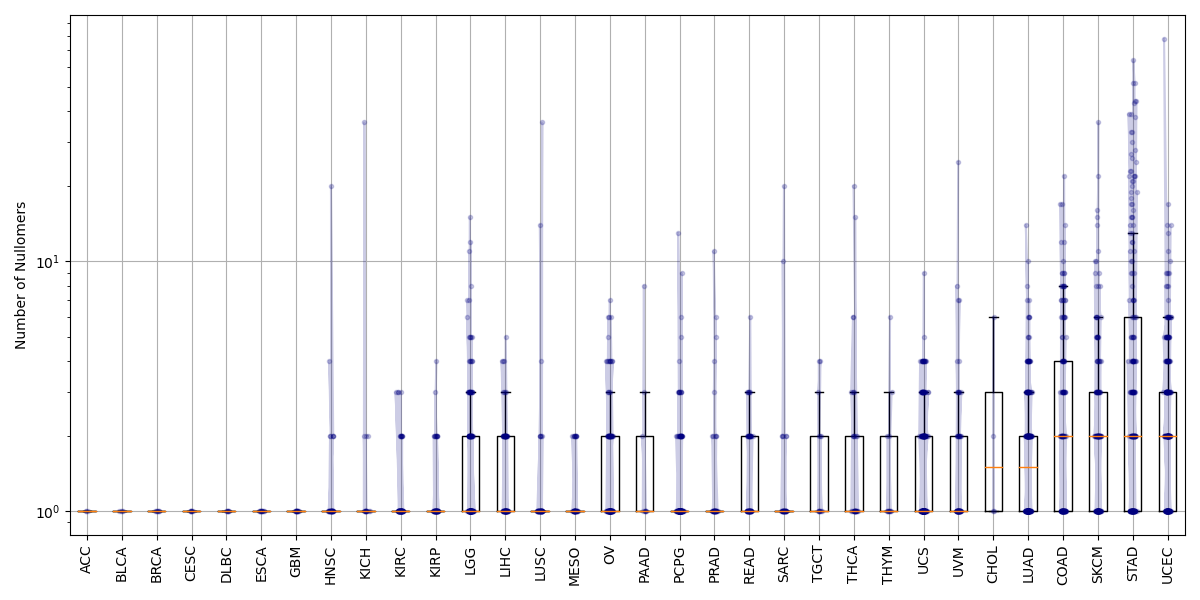
**

**B**

**
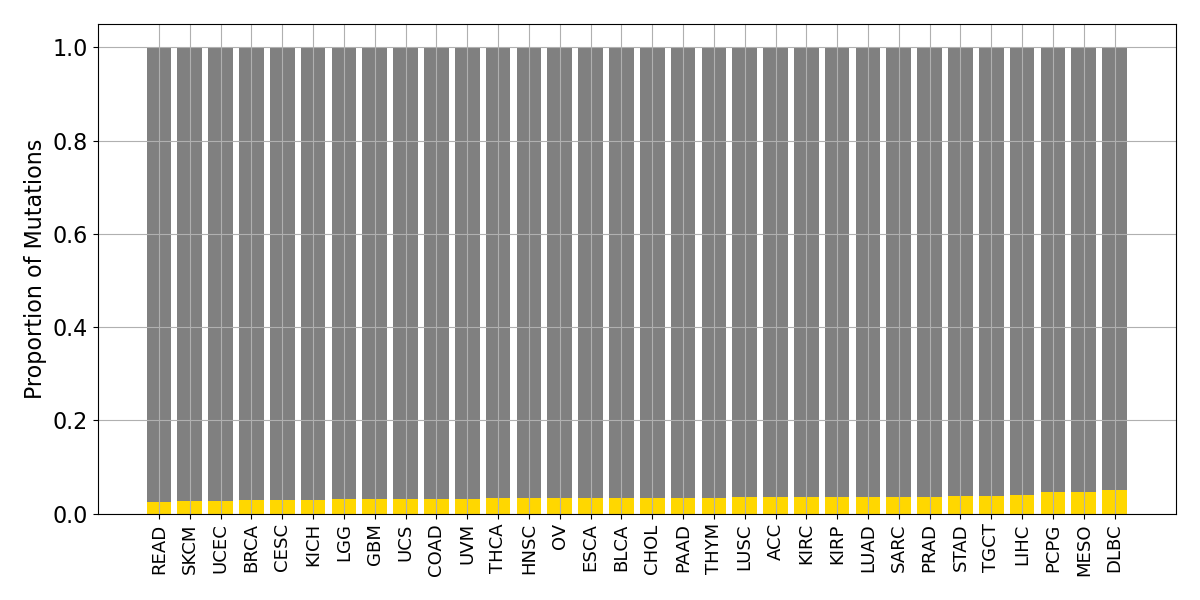

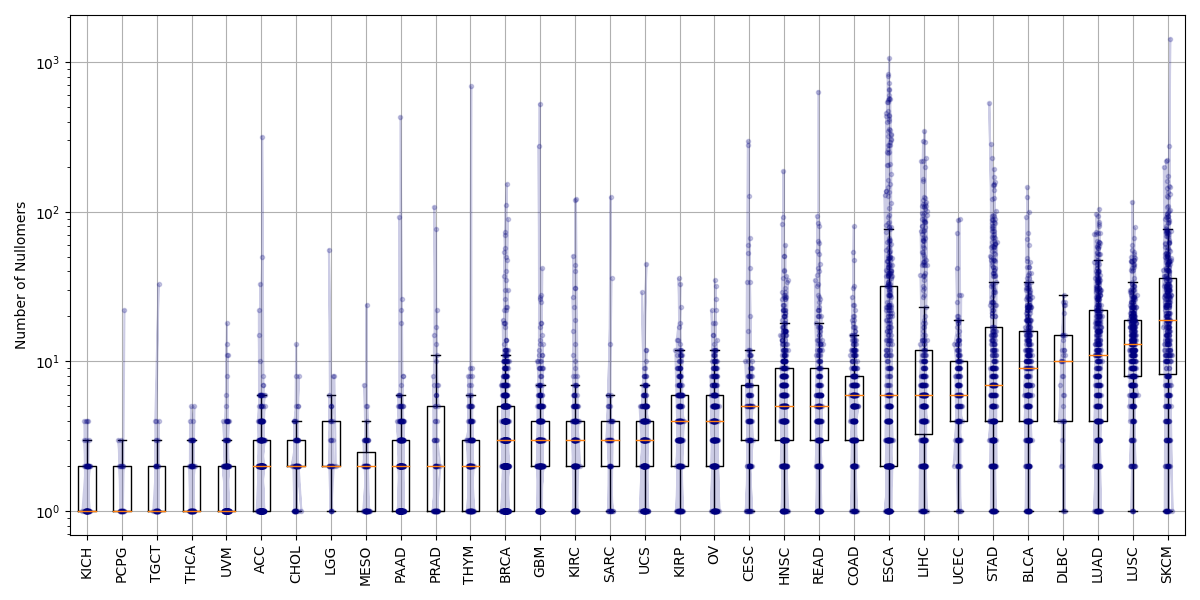
**

**C**

**
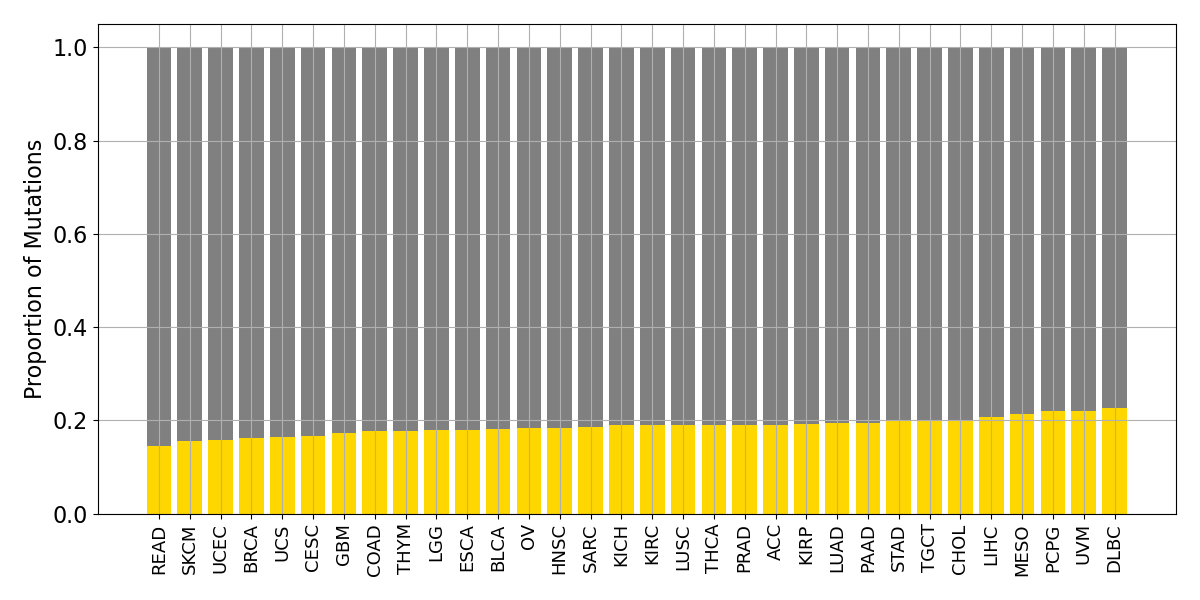

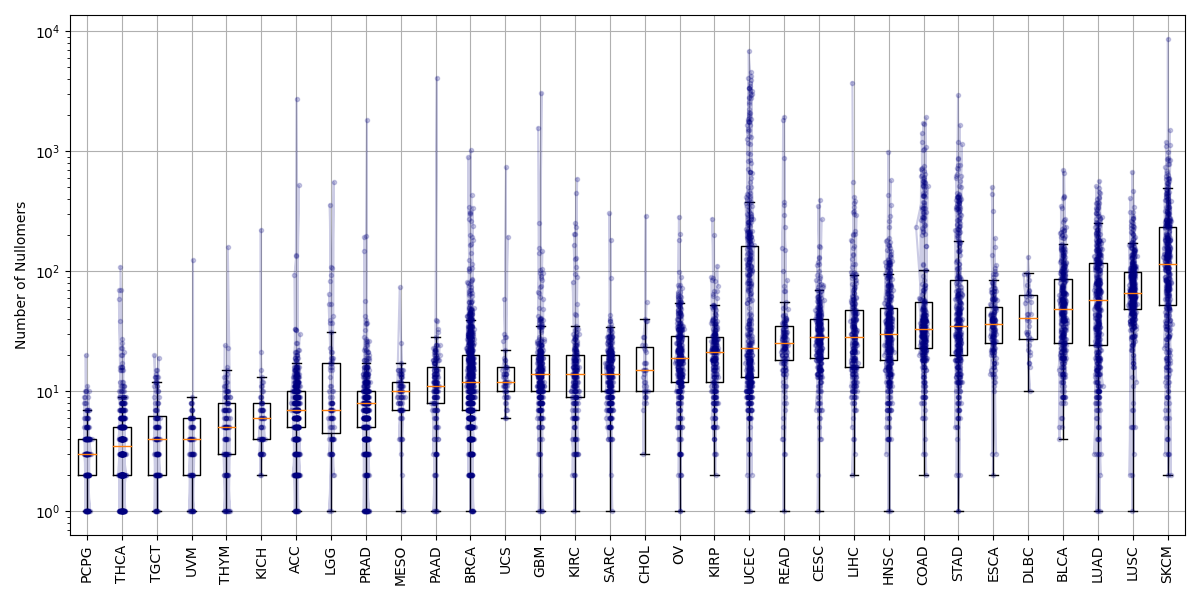
**

**D**

**
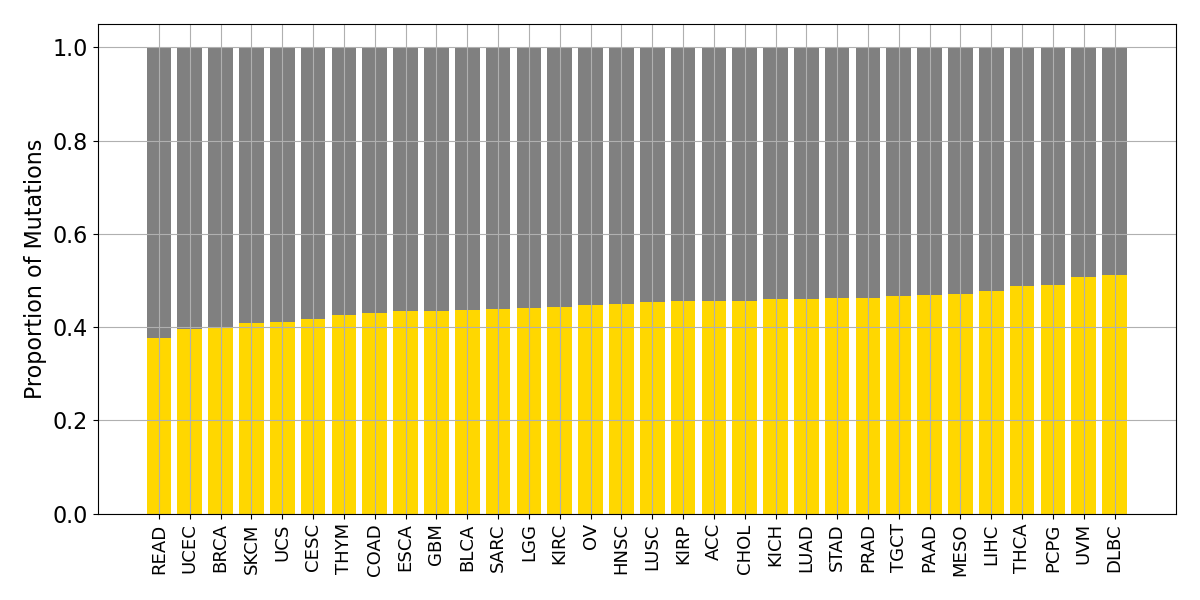

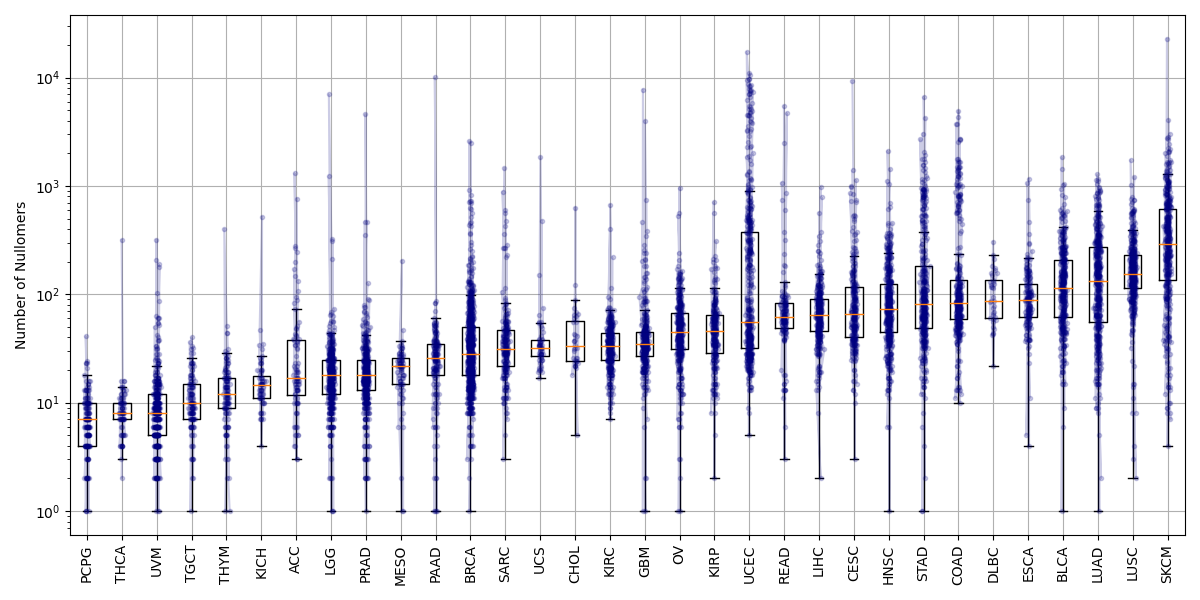
**

**Supplementary Figure 4: Proportion of mutations causing nullomer emergence across cancer types. Results shown for: a.** 12mers, **B.** 13mers, **C.** 14mers and **D.** 15mers.

**A B**

**
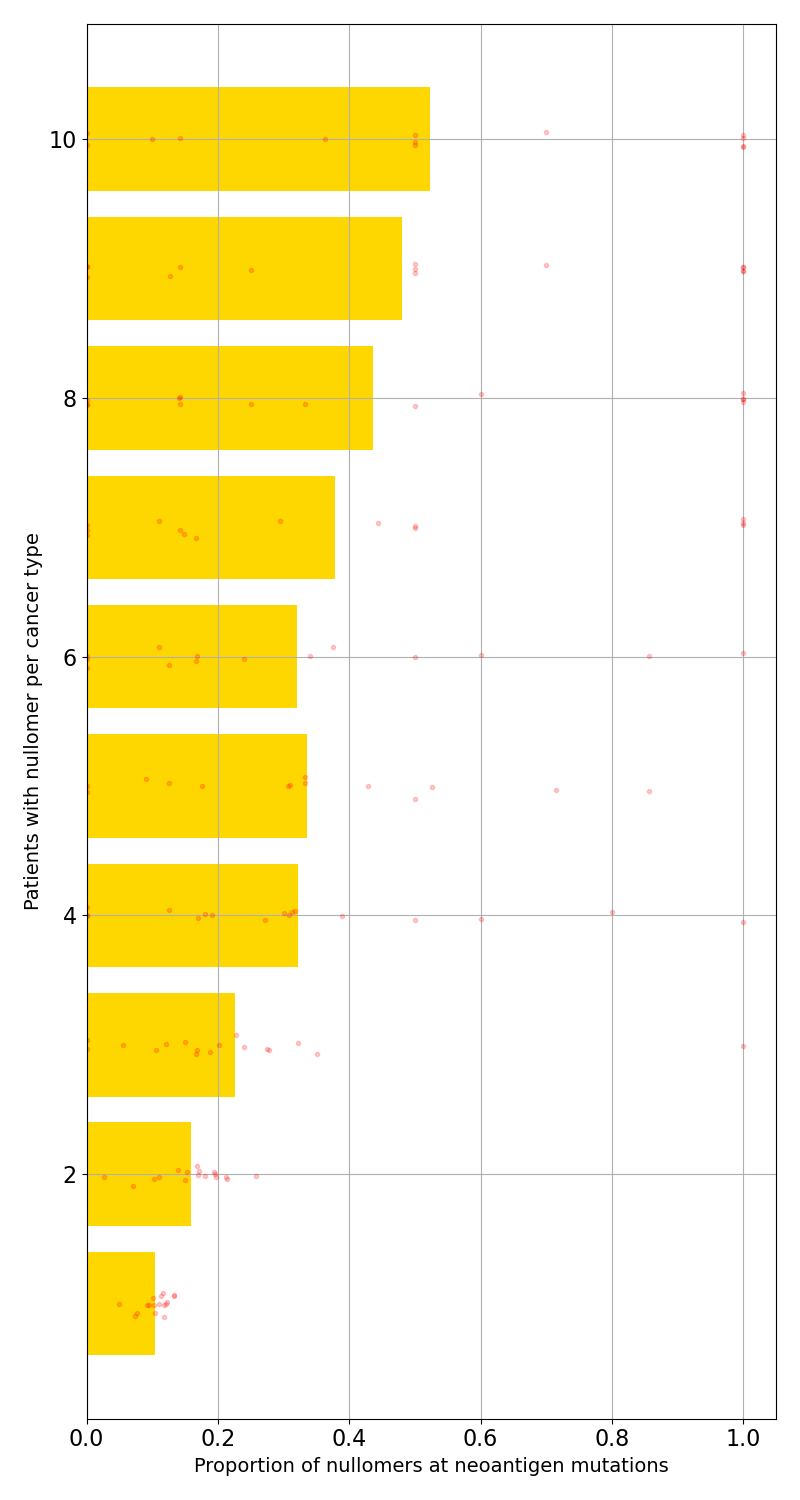

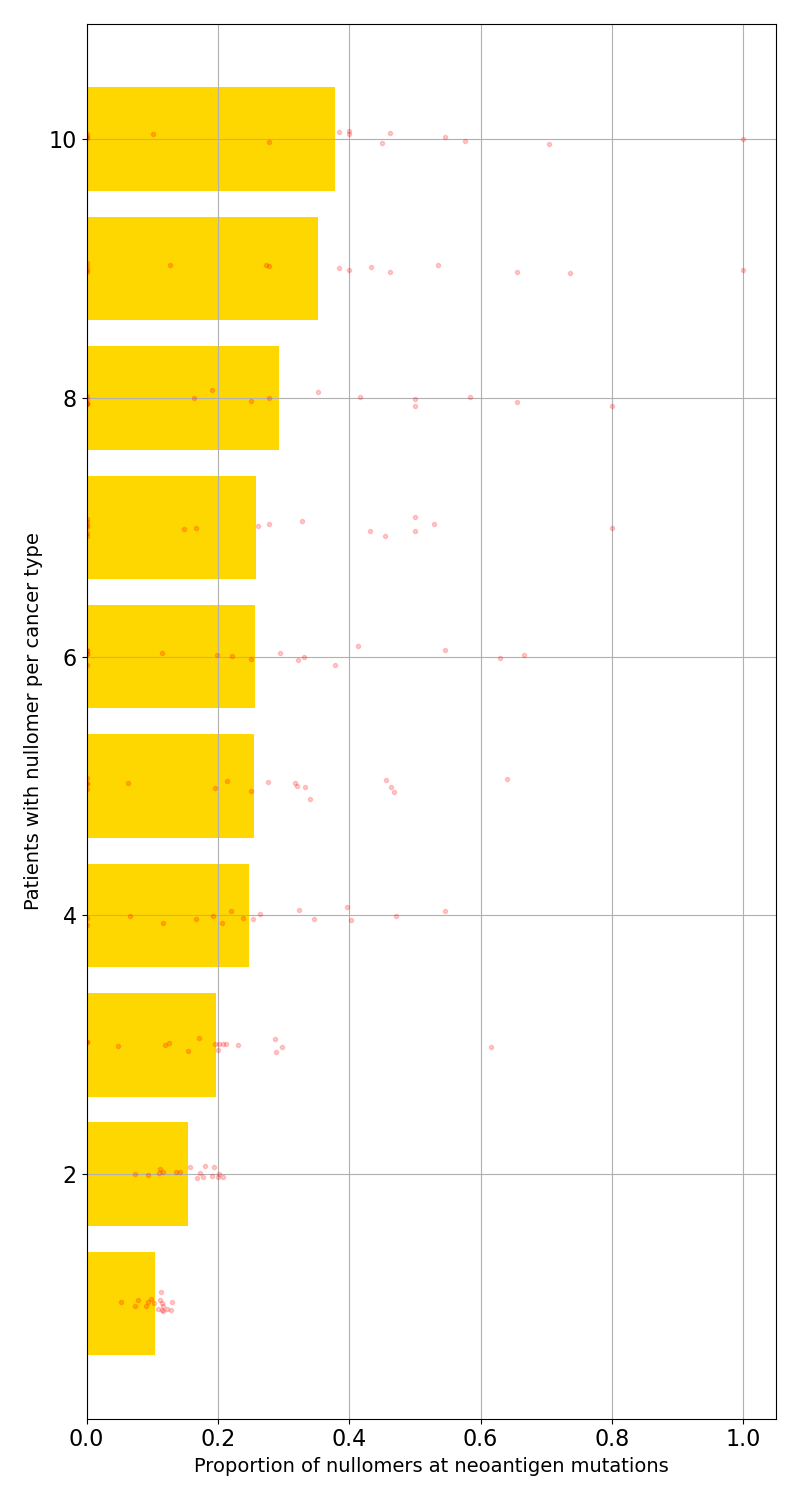
**

**Supplementary Figure 5: Association between the recurrence of nullomers in individual cancer types and the likelihood of them being derived from a neoantigen generating mutation.** Results shown for **A.** 14bp and **B.** 15bp nullomer lengths.

**
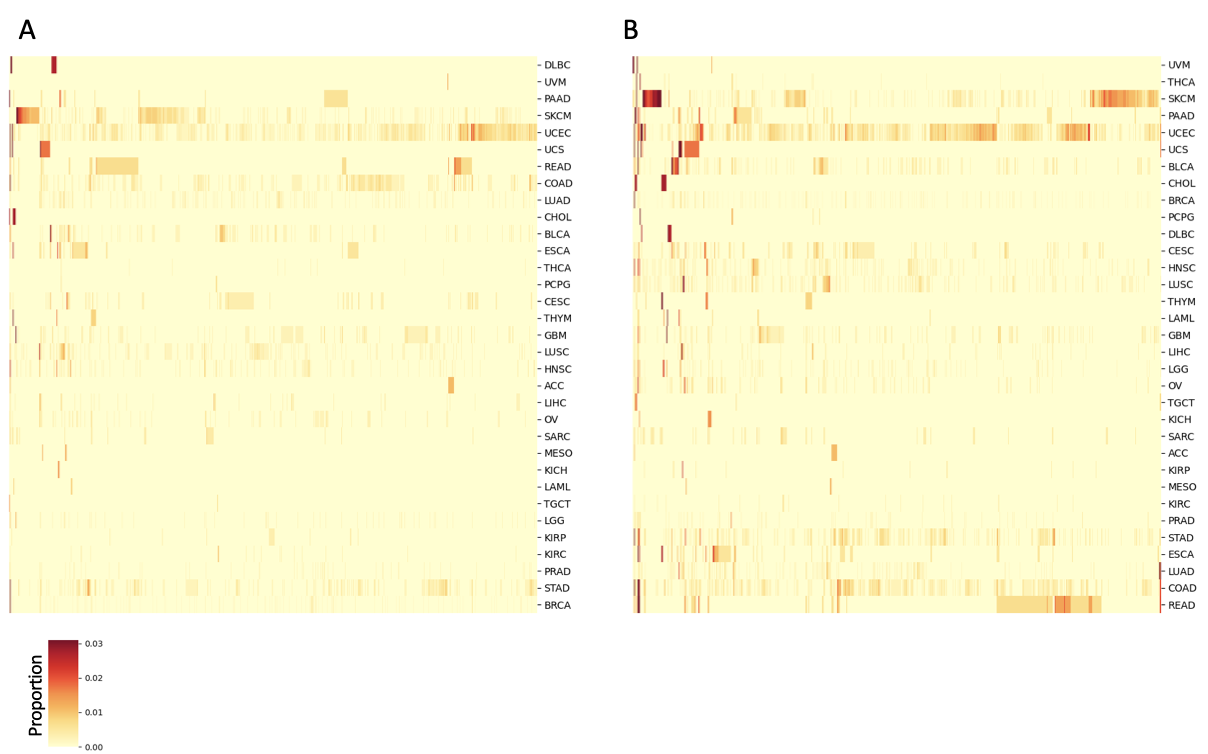
**

**Supplementary Figure 6: Proportion of patients in which each of the top 10,000 nullomers is found in for: A. 14bp nullomers, B. 15bp nullomers.**

**
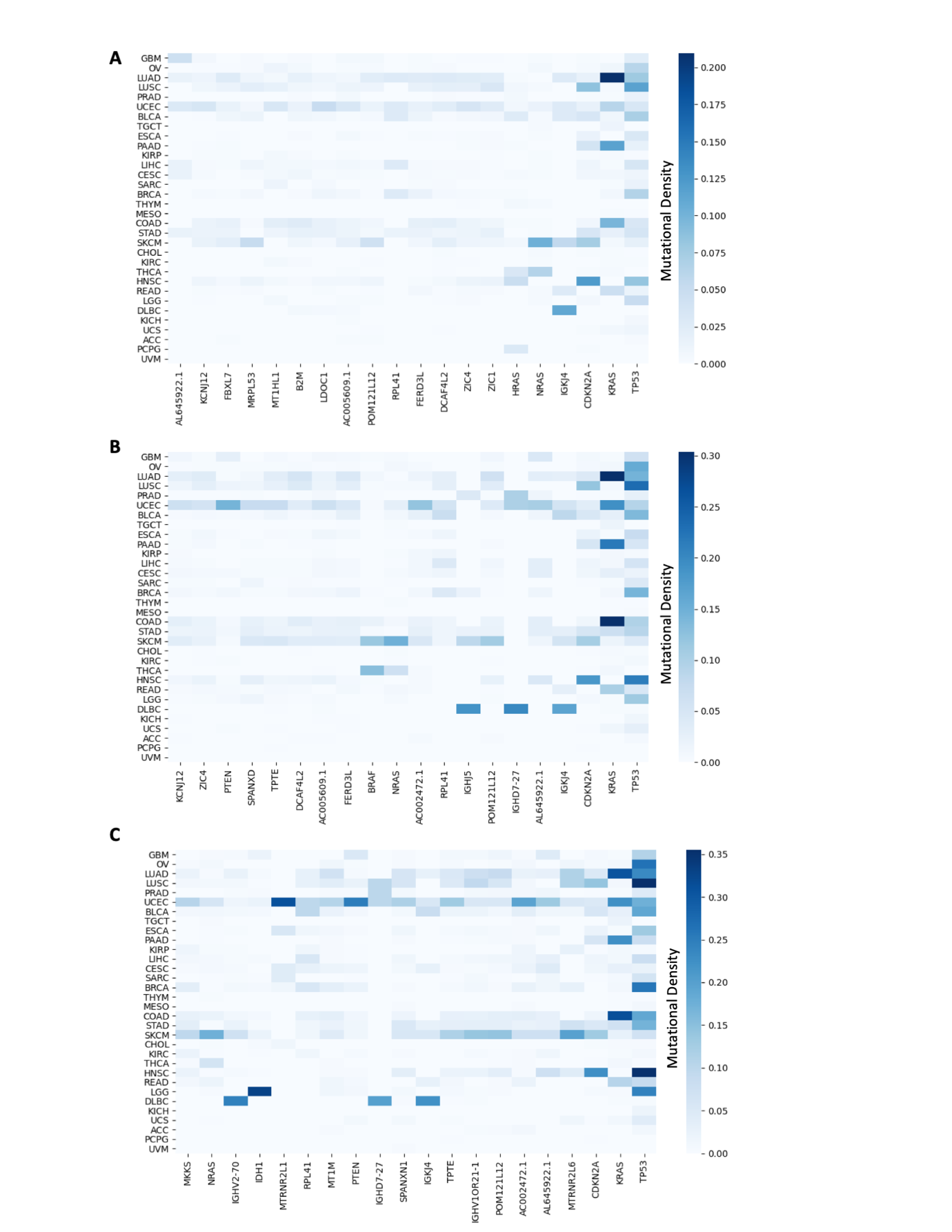
**

**Supplementary Figure 7: Nullomer emerging mutation density at coding regions across**

**cancer types. Results shown for: A.** 14-mers, **B.** 15-mers, **C.** 16-mers.

**A**


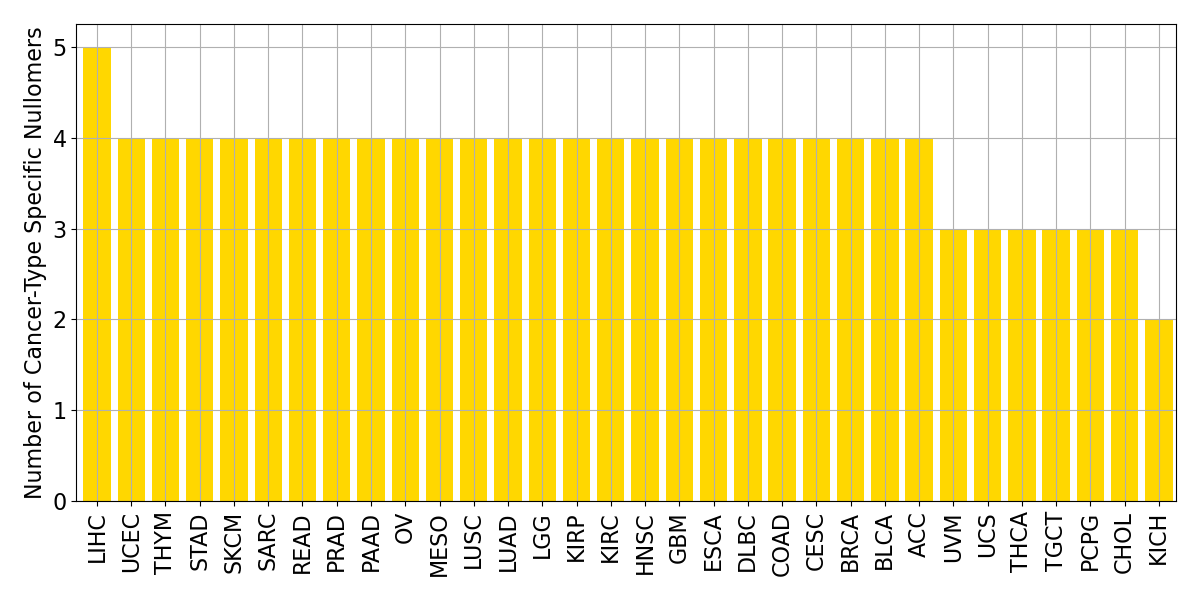


**B**


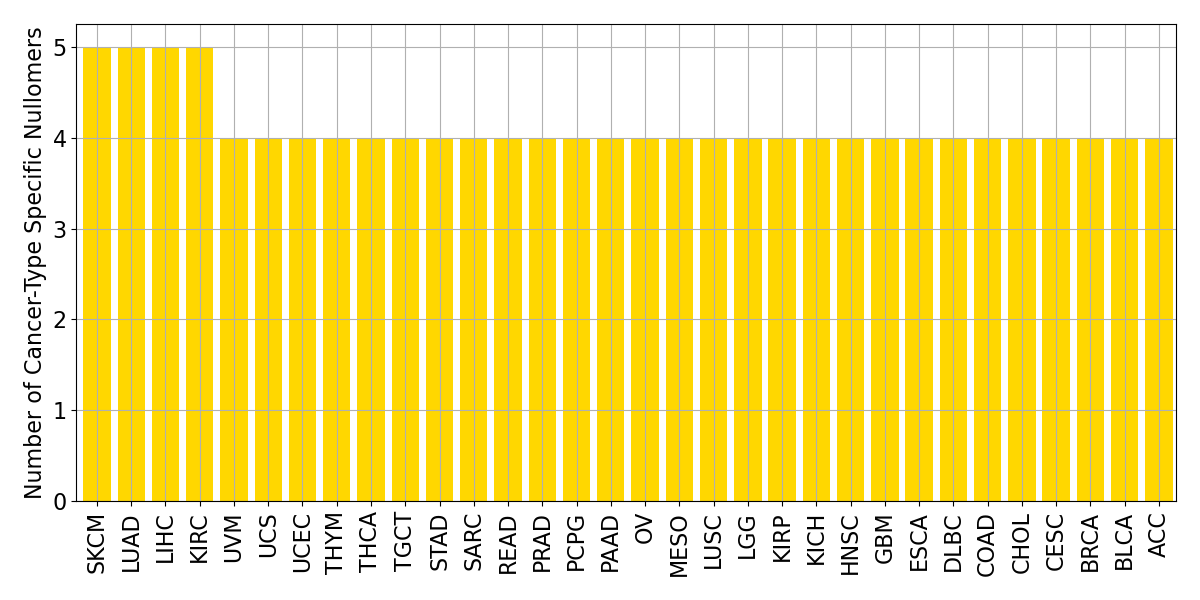


**C**


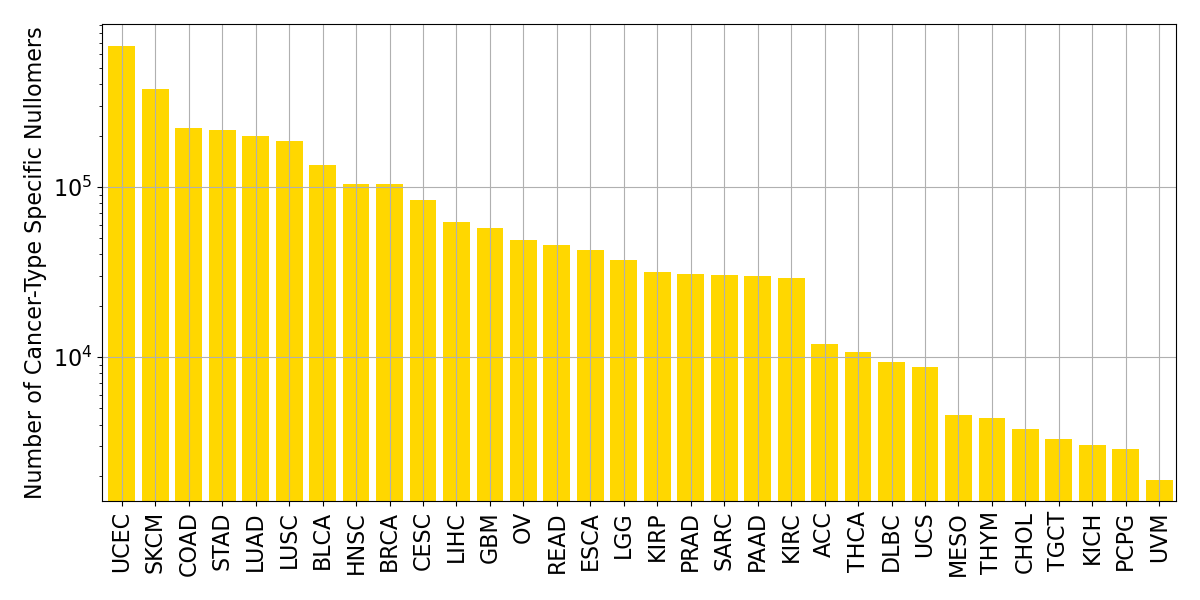


**D**


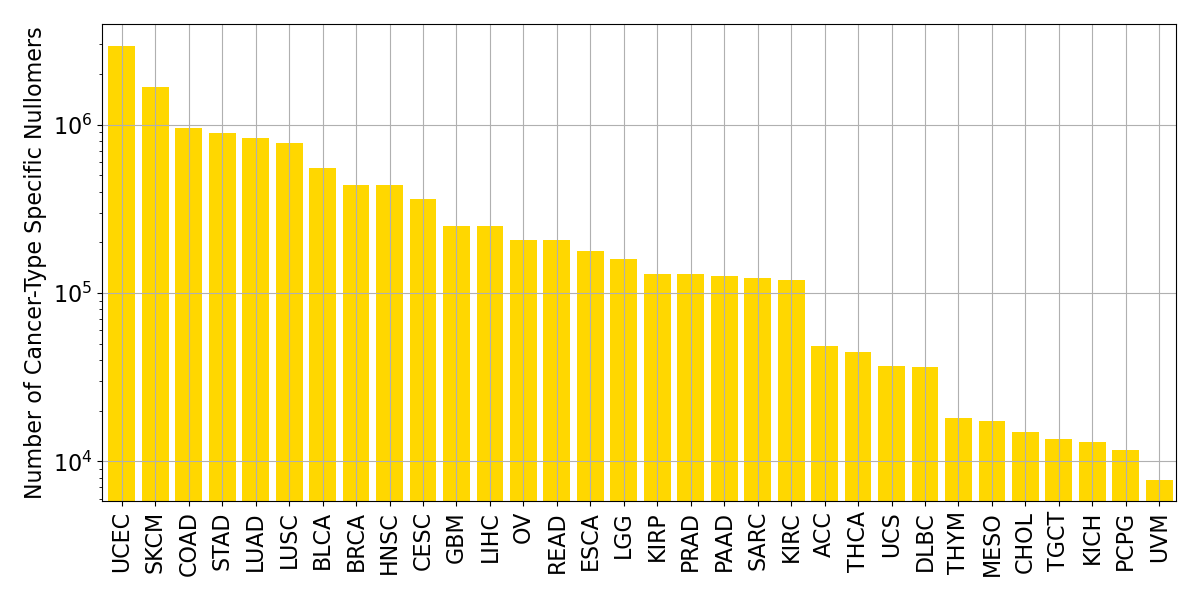


**Supplementary Figure 8: Identification of cancer-type specific nullomers. A.** Percentage of unique **A.** 12-mer, **B.** 13-mer, **C.** 14-mer and **D.** 15-mer nullomers per cancer type.


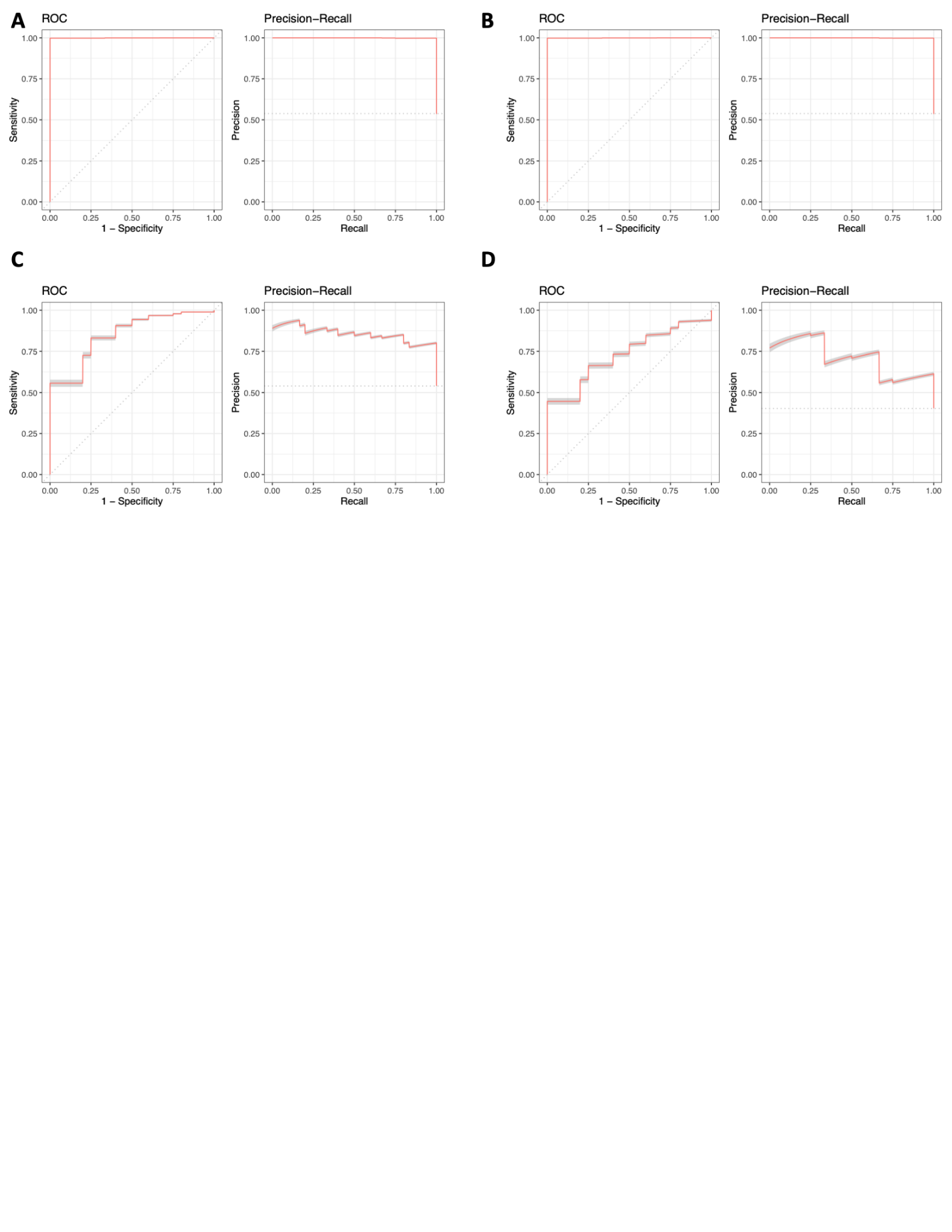


**Supplementary Figure 9: Predictive accuracy of cancer detection across cancer types.** ROC curve and precision recall for: **A.** HCC for 14mers, **B.** HCC for 15mers, C. colorectal cancer, **D.** esophageal cancer


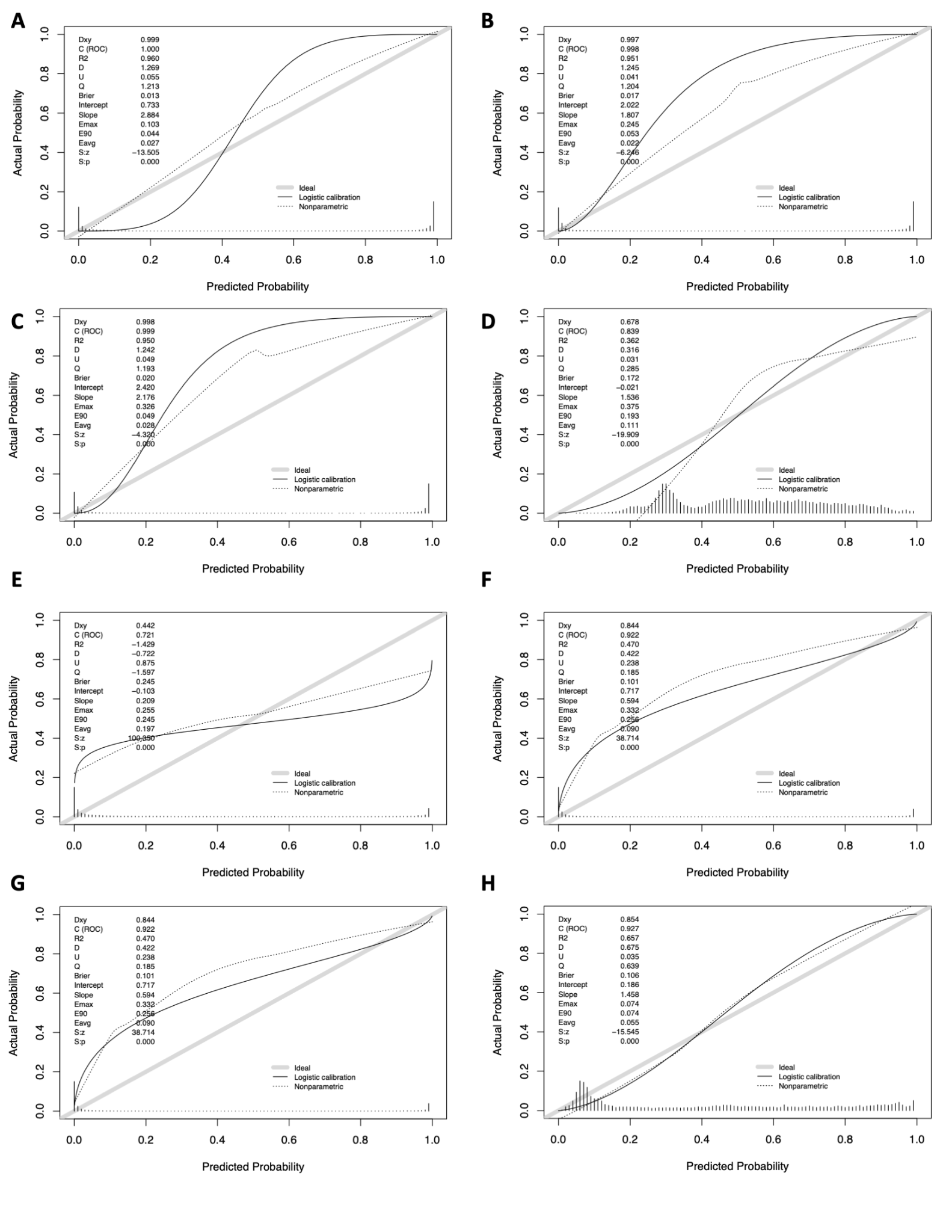


**Supplementary Figure 10: Calibration analysis to estimate performance of the cancer detection models.** Results shown for HCC cancer **A.-C.** using sixteen, fifteen-mer and fourteen-mer nullomers and **D.-H.**using sixteen-mer nullomers for **D.** colorectal, **E.** esophageal, **F.** lung, **G.** liver and **H.** stomach cancers.

**Supplementary Table 1: Up to fifteen highly frequent nullomers detected in selected top cancer genes for different kmer lengths.**

| gene | length:14 | length:15 | length:16 |
| --- | --- | --- | --- |
| *TP53* | CACTCGGATAAGAC,ACTCGGATAAGACG,TCGGATAAGACGCT,AGCGTCTTATCCGA,CGTCTTATCCGAGT,GTCTTATCCGAGTG,GCGCGGAAGCGGGT,ACCCGCTTCCGCGC,ATGAACCGGAGTCC,GAACCGGAGTCCCA,TGGGACTCCGGTTC,GGACTCCGGTTCAT,CCATGGCGAGGACG,CGTCCTCGCCATGG,CCGGCGCACAAAGG | GCATGAACCAGAGGC,GCCTCTGGTTCATGC,TGGGCGGCATGAACT,GGGCGGCATGAACTG,CAGTTCATGCCGCCC,AGTTCATGCCGCCCA,CCTCTGTGCGCCAGT,CTCTGTGCGCCAGTC,TCTGTGCGCCAGTCT,AGACTGGCGCACAGA,GACTGGCGCACAGAG,ACTGGCGCACAGAGG,TGAGCAGCGCTCACG,AGCAGCGCTCACGGT,GCAGCGCTCACGGTG | CGGAGGTTGTGAGGCA,GGAGGTTGTGAGGCAC,GAGGTTGTGAGGCACT,AGGTTGTGAGGCACTG,GGTTGTGAGGCACTGC,GTGGGGGCAGTGCCTC,GAGGCACTGCCCCCAC,GCAGTGCCTCACAACC,CAGTGCCTCACAACCT,AGTGCCTCACAACCTC,GTGCCTCACAACCTCC,TGCCTCACAACCTCCG,AACAGCTTTGAGGTGT,ACACCTCAAAGCTGTT,TGGGCGGCATGAACCA |
| *APC* | ATCGAACGACTCTA,TCGAACGACTCTAA,TTAGAGTCGTTCGA,TAGAGTCGTTCGAT,CCAGCTCCGTTTAG,CTCCGTTTAGAGTG,CACTCTAAACGGAG,CTAAACGGAGCTGG,CCCTCGATTTAATC,GATTAAATCGAGGG,GCAAAGTTCACGAC,GTCGTGAACTTTGC,TTCGCTCCTCAAGA,TCTTGAGGAGCGAA,GATGTAATTAGACG | ACATACTTCGTATAT,CATACTTCGTATATG,AAGCTGTCATATACG,CGTATATGACAGCTT,CATATACGAAGTATG,ATATACGAAGTATGT,GTAAAAAGACGTTGT,AACTTCTCACAACGT,ACGTTGTGAGAAGTT,ACAACGTCTTTTTAC,CATCATGTCAATTGG,CCAATTGACATGATG,AATCGAACGACTCTA,ATCGAACGACTCTAA,TCGAACGACTCTAAA | TTAGAACCCACTCAAT,ATTGAGTGGGTTCTAA,TGGCTATTCTTCACTA,TAGTGAAGAATAGCCA,GTATTACACTAAGATG,TCCAGCATATCATCTT,CACTAAGATGATATGC,AGCATATCATCTTAGT,ACTAAGATGATATGCT,GCATATCATCTTAGTG,AAGATGATATGCTGGA,CATCTTAGTGTAATAC,GACATACTTCGTATAT,ACATACTTCGTATATG,CATACTTCGTATATGA |
| *BRCA1* | CGTTCTAAATGACG,CTAAATGACGTAGA,TCTACGTCATTTAG,CGTCATTTAGAACG,GTTGTAGGTTTCCG,CGGAAACCTACAAC,CGTCCAATAAATCA,TGATTTATTGGACG,GCTCTTCGCGTTCA,CTCTTCGCGTTCAA,TCTTCGCGTTCAAG,CTTCGCGTTCAAGA,TTCGCGTTCAAGAA,TCGCGTTCAAGAAG,CTTCTTGAACGCGA | TTGCTCGCTTTGAAC,TCGCTTTGAACCTTG,CAAGGTTCAAAGCGA,GTTCAAAGCGAGCAA,GTAGTCATGCATCTT,AAGATGCATGACTAC,GCCACACGATTTGAT,CCACACGATTTGATG,CATCAAATCGTGTGG,ATCAAATCGTGTGGC,GACGTTCTAAATGAC,ACGTTCTAAATGACG,ATATTCATCTACGTC,CGTTCTAAATGACGT,GTTCTAAATGACGTA | CTTGCTCGCTTTGAAC,TTGCTCGCTTTGAACC,CTCGCTTTGAACCTTG,TCGCTTTGAACCTTGG,ACCAAGGTTCAAAGCG,CGCTTTGAACCTTGGT,CCAAGGTTCAAAGCGA,CAAGGTTCAAAGCGAG,GGTTCAAAGCGAGCAA,GTTCAAAGCGAGCAAG,TCTTGGCCCCTCTTTG,GCTCAGGGTTACCAAA,CTCAGGGTTACCAAAG,GGCCCCTCTTTGGTAA,GCCCCTCTTTGGTAAC |
| *BRCA2* | GGACAACCCGAATG,CATTCGGGTTGTCC,CCCGAACGTGATCA,CCGAACGTGATCAA,TTGATCACGTTCGG,TGATCACGTTCGGG,GTATTACCATGACG,CGTCATGGTAATAC,TACCGAAGGACCAA,TTGGTCCTTCGGTA,ATATACCTCATCCG,CGGATGAGGTATAT,TCACCTACGACTAG,ACCTACGACTAGAC,GTCTAGTCGTAGGT | CAACGAGAATAAATA,TATTTATTCTCGTTG,TGGATCCACACCTGA,TCAGGTGTGGATCCA,GGCAACAGCTTAACG,GCAACAGCTTAACGT,CAACAGCTTAACGTT,AACGTTAAGCTGTTG,ACGTTAAGCTGTTGC,CGTTAAGCTGTTGCC,AGGAATATCGTATGT,ACATACGATATTCCT,TATTTGCGCTCAATT,ATTTGCGCTCAATTA,TGCGCTCAATTAAAT | TTGAGCTTTCACAACT,AGTTGTGAAAGCTCAA,TCAATAGCTGACAAAG,CTTTGTCAGCTATTGA,CAAGCAAGTATTTTCC,GGAAAATACTTGCTTG,AAAGTGTCACTTGTTC,GTGTCACTTGTTCAGA,TGTCACTTGTTCAGAA,GTCACTTGTTCAGAAC,GAATGTTCTGAACAAG,CACTTGTTCAGAACAT,ATGTTCTGAACAAGTG,CTTGTTCAGAACATTC,GTTCTGAACAAGTGAC |
| *ATM* | CGGCAATATTATGA,TCATAATATTGCCG,GGATCGTAAATGCT,AGCATTTACGATCC,TGGTCACAACGATA,TATCGTTGTGACCA,AAGCGCCTGATTTG,CAAATCAGGCGCTT,GGTTTCAGATAGCG,GTACTTCAGGCGCT,TTTCAGATAGCGCC,GGCGCTATCTGAAA,AGCGCCTGAAGTAC,CGCTATCTGAAACC,TAAGCGCAGCCTTC | ACTTTCGAATTTGAG,ACACACTCAAATTCG,CGAATTTGAGTGTGT,CTCAAATTCGAAAGT,ACGGCAATATTATGA,CGGCAATATTATGAA,TTCATAATATTGCCG,TCATAATATTGCCGT,CACACTCGAATTTGA,TCAAATTCGAGTGTG,ACCTCCTGCTAAGCA,TGCTTAGCAGGAGGT,TCGCTTAGCAGGAAG,CTTCCTGCTAAGCGA,ATTCACTTGTACACC | TGACGGCAATATTACA,CGGCAATATTACAAAA,CAATATTACAAAATCC,GGATTTTGTAATATTG,TTTTGTAATATTGCCG,TGTAATATTGCCGTCA,GTCAACTTTCGAATTT,TCAACTTTCGAATTTG,CAACTTTCGAATTTGA,AACTTTCGAATTTGAG,ACTTTCGAATTTGAGT,CTTTCGAATTTGAGTG,TTTCGAATTTGAGTGT,CACACACTCAAATTCG,TTCGAATTTGAGTGTG |
| *KRAS* | TTGCCTACGCCAAC,GTTGGCGTAGGCAA,TACGCCACAAGCTC,GAGCTTGTGGCGTA,TGCCTACGCCACGA,GTAGTTGGAGCTCG,GCCTACGCCACGAG,TAGTTGGAGCTCGT,CCTACGCCACGAGC,CTACGCCACGAGCT,AGCTCGTGGCGTAG,GCTCGTGGCGTAGG,ACGAGCTCCAACTA,CTCGTGGCGTAGGC,CGAGCTCCAACTAC | CTTGCCTACGCCATC,TTGCCTACGCCATCA,TGATGGCGTAGGCAA,GATGGCGTAGGCAAG,TCTTGCCTACGCCAA,CTTGCCTACGCCAAC,TTGCCTACGCCAACA,TGTTGGCGTAGGCAA,GTTGGCGTAGGCAAG,TTGGCGTAGGCAAGA,CTTGCCTACGCCACA,TTGCCTACGCCACAA,GCCTACGCCACAAGC,CCTACGCCACAAGCT,CTACGCCACAAGCTC | TCTTGCCTACGCCATC,GGTAGTTGGAGCTGAT,CTTGCCTACGCCATCA,TTGCCTACGCCATCAG,AGTTGGAGCTGATGGC,GCCTACGCCATCAGCT,CCTACGCCATCAGCTC,TGGAGCTGATGGCGTA,TACGCCATCAGCTCCA,GAGCTGATGGCGTAGG,AGCTGATGGCGTAGGC,GCCATCAGCTCCAACT,CTGATGGCGTAGGCAA,TGATGGCGTAGGCAAG,ATCAGCTCCAACTACC |
| *PIK3CA* | GAATGATGCACGTC,GACGTGCATCATTC,GCCAATGGACGGTG,CCAATGGACGGTGT,ACACCGTCCATTGG,CACCGTCCATTGGC,GGTCTTTCGAATGC,GCATTCGAAAGACC,TATCAATGTTTCGA,TCGAAACATTGATA,ATTCGAAAGGCCCT,TCGAAAGGCCCTAG,CTAGGGCCTTTCGA,AGGGCCTTTCGAAT,ATTCGAGGCATTGA | TGAATGATGCACGTC,CAGCCACCATGACGT,GAATGATGCACGTCA,TGATGCACGTCATGG,CCATGACGTGCATCA,TGACGTGCATCATTC,ACGTCATGGTGGCTG,GACGTGCATCATTCA,AACCTACGTGAAAGT,ACTTTCACGTAGGTT,GCCGATAGCAAAATC,GATTTTGCTATCGGC,CCATGCCAATGGACG,ATGCCAATGGACGGT,TTAAGGAACACCGTC | TGAAATCACTAAGCAG,CTGCTTAGTGATTTCA,GTCCAGCCACCATGAC,TCCAGCCACCATGACG,ATGAATGATGCACGTC,CCAGCCACCATGACGT,TGAATGATGCACGTCA,CAGCCACCATGACGTG,GAATGATGCACGTCAT,ATGATGCACGTCATGG,CCACCATGACGTGCAT,TGATGCACGTCATGGT,CACCATGACGTGCATC,GATGCACGTCATGGTG,ACCATGACGTGCATCA |
| *LRP1B* | CGGCTAGAATCAAT,ATTGATTCTAGCCG,TAGATGGGCGGCAC,GTGCCGCCCATCTA,GCGGAAATGGGTGT,ACACCCATTTCCGC,ATGGAATCGATACG,TGGAATCGATACGC,ACTGTGGCGTATCG,ATCGATACGCCACA,TGTGGCGTATCGAT,CGATACGCCACAGT,GCGTATCGATTCCA,CGTATCGATTCCAT,TGGATATGCGACGT | GATAGACTTTGATGC,GCATCAAAGTCTATC,GGTCGGCTAGAATCA,TCGGCTAGAATCAAT,CGGCTAGAATCAATC,GGCTAGAATCAATCC,GGATTGATTCTAGCC,GATTGATTCTAGCCG,ATTGATTCTAGCCGA,TGATTCTAGCCGACC,GTAGATGGGCGGCAC,TAGATGGGCGGCACT,AGTGCCGCCCATCTA,GTGCCGCCCATCTAC,CGGTTTATCCTTACA | TCAGATGCATCAAAGT,CAGATGCATCAAAGTC,TGATAGACTTTGATGC,GATAGACTTTGATGCA,TGCATCAAAGTCTATC,GCATCAAAGTCTATCA,GACTTTGATGCATCTG,ACTTTGATGCATCTGA,GCCACTAACCCAATTT,CCACTAACCCAATTTG,CAAATTGGGTTAGTGG,AAATTGGGTTAGTGGC,TACTGGACTGATGGAA,TTCCATCAGTCCAGTA,GGAATATCGGAACAAA |
| *APC* | ATCGAACGACTCTA,TCGAACGACTCTAA,TTAGAGTCGTTCGA,TAGAGTCGTTCGAT,CCAGCTCCGTTTAG,CTCCGTTTAGAGTG,CACTCTAAACGGAG,CTAAACGGAGCTGG,CCCTCGATTTAATC,GATTAAATCGAGGG,GCAAAGTTCACGAC,GTCGTGAACTTTGC,TTCGCTCCTCAAGA,TCTTGAGGAGCGAA,GATGTAATTAGACG | ACATACTTCGTATAT,CATACTTCGTATATG,AAGCTGTCATATACG,CGTATATGACAGCTT,CATATACGAAGTATG,ATATACGAAGTATGT,GTAAAAAGACGTTGT,AACTTCTCACAACGT,ACGTTGTGAGAAGTT,ACAACGTCTTTTTAC,CATCATGTCAATTGG,CCAATTGACATGATG,AATCGAACGACTCTA,ATCGAACGACTCTAA,TCGAACGACTCTAAA | TTAGAACCCACTCAAT,ATTGAGTGGGTTCTAA,TGGCTATTCTTCACTA,TAGTGAAGAATAGCCA,GTATTACACTAAGATG,TCCAGCATATCATCTT,CACTAAGATGATATGC,AGCATATCATCTTAGT,ACTAAGATGATATGCT,GCATATCATCTTAGTG,AAGATGATATGCTGGA,CATCTTAGTGTAATAC,GACATACTTCGTATAT,ACATACTTCGTATATG,CATACTTCGTATATGA |
| *FAT4* | AGTTTTCGCATCAA,TTTCGCATCAACCC,TTCGCATCAACCCA,TGGGTTGATGCGAA,GGGTTGATGCGAAA,TTGATGCGAAAACT,GGTACGCCGAGACA,GTACGCCGAGACAA,TACGCCGAGACAAA,CGCCGAGACAAAGT,ACTTTGTCTCGGCG,TTTGTCTCGGCGTA,TTGTCTCGGCGTAC,TGTCTCGGCGTACC,GTGTAACCAGTGCG | ATAGTTTTCGCATCA,TAGTTTTCGCATCAA,AGTTTTCGCATCAAC,CGGATTCTGGGTTGA,GTTTTCGCATCAACC,TTTTCGCATCAACCC,TTTCGCATCAACCCA,TTCGCATCAACCCAG,TTCTGGGTTGATGCG,CGCATCAACCCAGAA,CTGGGTTGATGCGAA,TGGGTTGATGCGAAA,GGGTTGATGCGAAAA,GGTTGATGCGAAAAC,TCAACCCAGAATCCG | TTCACTGCTAGTTTGT,TCACTGCTAGTTTGTG,CACTGCTAGTTTGTGC,ATCAGCACAAACTAGC,GCTAGTTTGTGCTGAT,GCACAAACTAGCAGTG,CACAAACTAGCAGTGA,ACAAACTAGCAGTGAA,GATAGTTTTCGCATCA,CTCCGGATTCTGGGTT,ATAGTTTTCGCATCAA,TAGTTTTCGCATCAAC,CCGGATTCTGGGTTGA,AGTTTTCGCATCAACC,CGGATTCTGGGTTGAT |
| *BRAF* | GTTTGACAGCGCAA,CCAGGGTTTGCGCT,AGCGCAAACCCTGG,TTGCGCTGTCAAAC,CATTCGATTCCGGT,ATTCGATTCCGGTC,TTCGATTCCGGTCT,TCGATTCCGGTCTT,AAGACCGGAATCGA,AGACCGGAATCGAA,GACCGGAATCGAAT,ACCGGAATCGAATG,AACGAGACCGATCT,GACCGATCTTCATC,GATGAAGATCGGTC | CCATCGAGATTTCTC,CATCGAGATTTCTCT,AGAGAAATCTCGATG,GAGAAATCTCGATGG,CATCGAGATTTCATT,TCGAGATTTCATTGT,GTCTAGCTACAATGA,CGAGATTTCATTGTA,TACAATGAAATCTCG,TCATTGTAGCTAGAC,ACAATGAAATCTCGA,AATGAAATCTCGATG,TCCATCGAGATTCCA,CCATCGAGATTCCAC,CATCGAGATTCCACT | CTCCATCGAGATTTCT,TCCATCGAGATTTCTC,TGGTCTAGCTACAGAG,CCATCGAGATTTCTCT,CATCGAGATTTCTCTG,ATCGAGATTTCTCTGT,TCGAGATTTCTCTGTA,CGAGATTTCTCTGTAG,CTACAGAGAAATCTCG,TACAGAGAAATCTCGA,ACAGAGAAATCTCGAT,CAGAGAAATCTCGATG,AGAGAAATCTCGATGG,CTCTGTAGCTAGACCA,GAGAAATCTCGATGGA |
| *PTEN* | GACCCACATGACGG,CCGTCATGTGGGTC,TGATCAATAAATCG,CGATTTATTGATCA,GGTAACGGCTAAGG,GTAACGGCTAAGGG,TAACGGCTAAGGGA,TCCCTTAGCCGTTA,CCCTTAGCCGTTAC,CCTTAGCCGTTACC,TACACCAGTTCGTT,AACGAACTGGTGTA,ATAGCGCCTCTGAT,ATCAGAGGCGCTAT,TGAAGACCGTAACC | TACACCAGTTTGTCC,GGACAAACTGGTGTA,TACACCAGTTCCTCC,GGAGGAACTGGTGTA,AGGACCCACATGACG,GGACCCACATGACGG,GACCCACATGACGGG,CCCGTCATGTGGGTC,CCGTCATGTGGGTCC,CGTCATGTGGGTCCT,CTATCGATTTATTGA,TATCGATTTATTGAT,GTGATCAATAAATCG,TGATCAATAAATCGA,TCGATTTATTGATCA | TCATTACACCAGTTTG,CATTACACCAGTTTGT,TTACACCAGTTTGTCC,TACACCAGTTTGTCCC,GGGACAAACTGGTGTA,GGACAAACTGGTGTAA,ACAAACTGGTGTAATG,CAAACTGGTGTAATGA,ATTACACCAGTTCCTC,TTACACCAGTTCCTCC,TACACCAGTTCCTCCC,GGGAGGAACTGGTGTA,GGAGGAACTGGTGTAA,GAGGAACTGGTGTAAT,ACTTGTCTTCCCGTCA |
| *MLH1* | CCGATACAAAGTGC,GCACTTTGTATCGG,TTCGAGGTTAGGTA,TACCTAACCTCGAA,GAAAGCCCGTCCTA,TAGGACGGGCTTTC,GTGAACCGCATTGC,TGAACCGCATTGCG,CCCGCCGCAATGCG,ACCGCATTGCGGCG,CCGCCGCAATGCGG,CCGCATTGCGGCGG,CGCCGCAATGCGGT,CGCATTGCGGCGGG,CGCAATGCGGTTCA | TACCAGACAATCTAA,TTAGATTGTCTGGTA,CGGGAATCTGTACAA,TTGTACAGATTCCCG,TCCGATACAAAGTGC,CCGATACAAAGTGCT,AGCACTTTGTATCGG,GCACTTTGTATCGGA,GACAATATTCGCTTC,ACAATATTCGCTTCA,TCCAAAGATGAAGCG,CCAAAGATGAAGCGA,TCGCTTCATCTTTGG,CGCTTCATCTTTGGA,TGAAGCGAATATTGT | TTGATTCTACCAGACA,GATTCTACCAGACAAT,CTACCAGACAATCTAA,GTTTAGATTGTCTGGT,TACCAGACAATCTAAA,TTTAGATTGTCTGGTA,ACCAGACAATCTAAAC,TTAGATTGTCTGGTAG,ATTGTCTGGTAGAATC,TGTCTGGTAGAATCAA,GCCCACCAGATGGTTT,CCGGGAATCTGTACAA,CGGGAATCTGTACAAA,ACCAGATGGTTTGTAC,ATCTGTACAAACCATC |
